# A Systematic Review of the Genotoxicity and Antigenotoxicity of Biologically Synthesized Metallic Nanomaterials: Are Green Nanoparticles Safe Enough for Clinical Marketing?

**DOI:** 10.3390/medicina55080439

**Published:** 2019-08-05

**Authors:** Hamed Barabadi, Masoud Najafi, Hadi Samadian, Asaad Azarnezhad, Hossein Vahidi, Mohammad Ali Mahjoub, Mahbobeh Koohiyan, Amirhossein Ahmadi

**Affiliations:** 1 Department of Pharmaceutical Biotechnology, School of Pharmacy, Shahid Beheshti University of Medical Sciences, Tehran 19968 35113, Iran; 2 Radiology and Nuclear Medicine Department, School of Paramedical Sciences, Kermanshah University of Medical Sciences, Kermanshah 671584 7141, Iran; 3 Nano Drug Delivery Research Center, Health Technology Institute, Kermanshah University of Medical Sciences, Kermanshah 67145 1673, Iran; 4 Pharmaceutical Sciences Research Center, Health Institute, Kermanshah University of Medical Sciences, Kermanshah 67145 1673, Iran; 5 Cellular and Molecular Research Center, Research Institute for Health Development, Kurdistan University of Medical Sciences, Sanandaj 66186 34683, Iran; 6 Department of Pharmaceutics, School of Pharmacy, Shahid Beheshti University of Medical Sciences, Tehran 19968 35113, Iran; 7 Cancer Research Center, Shahrekord University of Medical Sciences, Shahrekord 88138 33435, Iran; 8 Pharmaceutical Sciences Research Center, Faculty of Pharmacy, Mazandaran University of Medical Sciences, Sari 48175 861 Iran

**Keywords:** genotoxicity, biosynthesis, metal nanoparticles, systematic review

## Abstract

*Background and objectives:* Although studies have elucidated the significant biomedical potential of biogenic metallic nanoparticles (MNPs), it is very important to explore the hazards associated with the use of biogenic MNPs. Evidence indicates that genetic toxicity causes mutation, carcinogenesis, and cell death. *Materials and Methods:* Therefore, we systematically review original studies that investigated the genotoxic effect of biologically synthesized MNPs via in vitro and in vivo models. Articles were systematically collected by screening the literature published online in the following databases; Cochrane, Web of Science, PubMed, Scopus, Science Direct, ProQuest, and EBSCO. *Results*: Most of the studies were carried out on the MCF-7 cancer cell line and phytosynthesis was the general approach to MNP preparation in all studies. Fungi were the second most predominant resource applied for MNP synthesis. A total of 80.57% of the studies synthesized biogenic MNPs with sizes below 50 nm. The genotoxicity of Ag, Au, ZnO, TiO_2_, Se, Cu, Pt, Zn, Ag-Au, CdS, Fe_3_O_4_, Tb_2_O_3_, and Si-Ag NPs was evaluated. AgNPs, prepared in 68.79% of studies, and AuNPs, prepared in 12.76%, were the two most predominant biogenic MNPs synthesized and evaluated in the included articles. *Conclusions*: Although several studies reported the antigenotoxic influence of biogenic MNPs, most of them reported biogenic MNP genotoxicity at specific concentrations and with a dose or time dependence. To the best of our knowledge, this is the first study to systematically evaluate the genotoxicity of biologically synthesized MNPs and provide a valuable summary of genotoxicity data. In conclusion, our study implied that the genotoxicity of biologically synthesized MNPs varies case-by-case and highly dependent on the synthesis parameters, biological source, applied assay, etc. The gathered data are required for the translation of these nanoproducts from research laboratories to the clinical market.

## 1. Introduction

**Figure 1:**
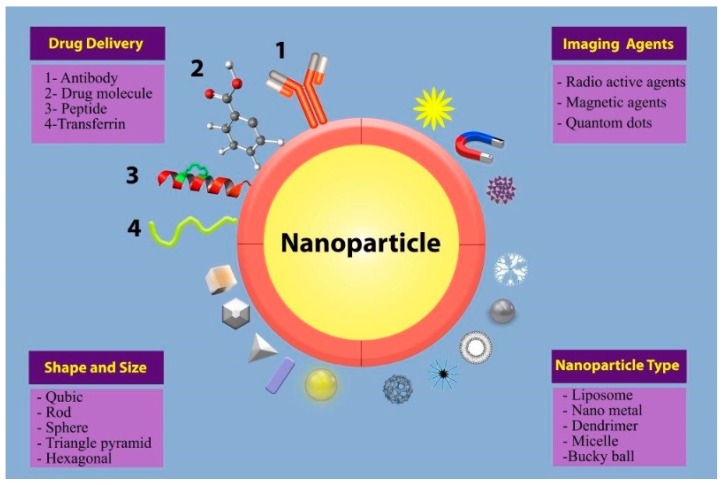
Schematic representation of nanoparticle types, shapes, and sizes, as well as drug delivery and imaging agents.

**Figure 2:**
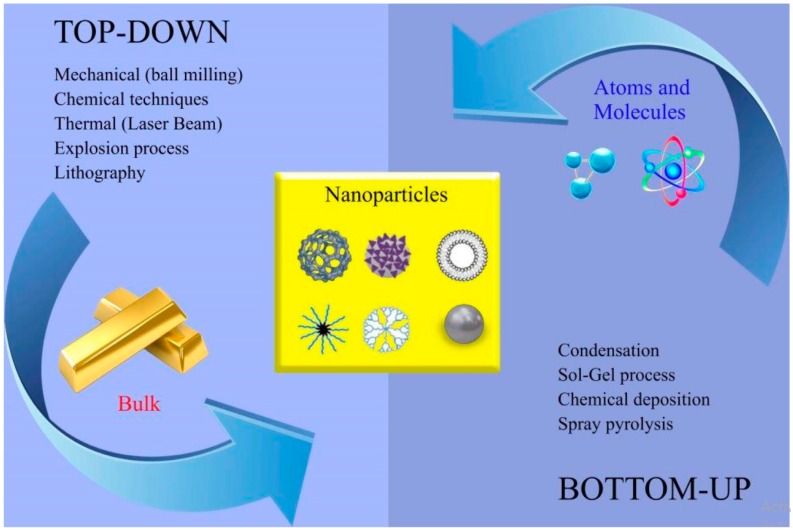
Top-down and bottom-up approaches exploiting different physical, chemical, and biological methods for the synthesis of nanoparticles (NPs).

Nanotechnology is a multidisciplinary and interdisciplinary science that can be defined as the design, synthesis, and application of materials and devices at the nanolevel in different areas, including medicine, biology, physics, chemistry, and engineering [1,2]. Different types of nanomaterials with a wide range of size and shape have been exploited in different fields of biomedicine such as drug delivery and imaging (Figure 1). The global nanotechnology industry is anticipated to reach US $75.8 billion by 2020 [3]. The synthesis of metal nanoparticles (MNPs) is an emerging area of interest in nanoscience due to their unique physical, chemical, electrical, and optical properties compared to those of the bulk material [2]. The global sale of MNPs is anticipated to reach US $50 billion by 2026 [4]. Generally, there are two approaches to the synthesis of MNPs: the top-down approach and the bottom-up approach. A schematic of these approaches is shown in Figure 2. In the top-down approach, MNPs are synthesized by cutting the bulk material via different mechanical procedures to produce nanosized structures. In the bottom-up approach, MNPs are synthesized at the molecular level via different chemical or biological procedures [3,5]. Among the physical, chemical, and biological methods for MNP synthesis, the biological methods are bottom-up approaches that overcome some of the unsatisfactory conditions of the physical and chemical methods (e.g., high energy or temperature requirements, hazardous waste generation, and toxic chemical use) [6,7,8]. Nanobiotechnology studies are categorized as “Gold Biotechnology” studies and involve the synthesis of MNPs by exploiting biological sources [7]. A number of plants [7,9,10,11], algae [12,13], fungi [14], and bacteria [15,16] have been reported to biosynthesize MNPs as ecofriendly, biocompatible, and inexpensive alternatives to conventional physical and chemical methods. Notably, MNPs have been used in wound dressings, cell labeling, photoimaging, sensors, drug delivery, gene delivery, photothermal therapy, etc. [7]. Additionally, a recent 2018 meta-analysis reported that the cytotoxicity of biogenic MNPs in cancer cell lines was 9 times higher than that in normal cell lines, indicating much greater MNP cytotoxicity in cancer cell lines (OR = 9.004, *p* ˂ 0.001) [16]. Although studies have elucidated the significant biomedical and pharmaceutical potential of biogenic MNPs, exploring the hazards associated with the use of biogenic MNPs is very important. The large surface area, high cellular penetration ability, and significant catalytic activity of MNPs may result in MNPs having greater toxicity than their bulk counterparts [17].

**Figure 3:**
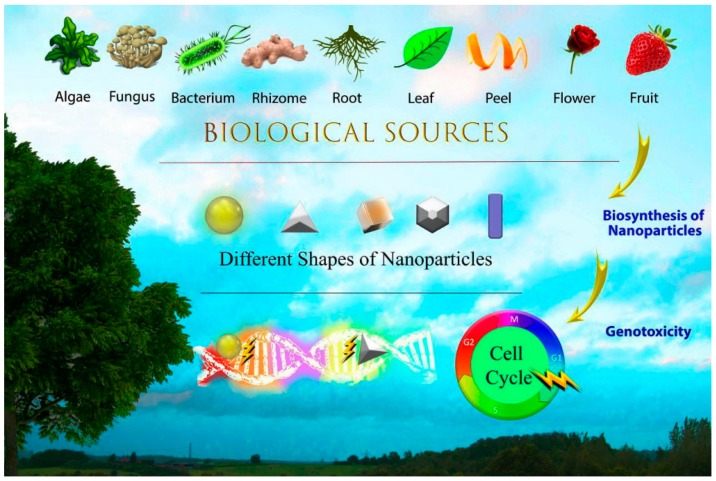
The interface of nature, nanotechnology, and genotoxicity.

Genotoxicity is a concern associated with the use of MNPs because it plays a major role in the initiation and progression of abnormalities. Genotoxicity can be defined as destructive genetic alterations involving gene mutations, structural chromosomal aberrations, and recombination that are induced by genotoxins [17,18,19]. Genotoxins are agents that can damage the DNA sequence and chromosomal structure by addition, deletion, duplication, ring formation, etc. Genotoxins are classified into three groups on the basis of their effects: (a) carcinogens or cancer-causing agents, (b) mutagens or mutation-causing agents, and (c) teratogens or birth-defect-causing agents [20]. Registering a new drug without performing genotoxicity assays is impossible. In 1984, the Ministry of Health and Welfare (MHW) of Japan adopted the use of genotoxicity assays to approve new drugs [21]. In addition, the FDA recommends genotoxicity testing for all new drugs before carrying out clinical trials and receiving marketing authorization. The regulatory authorities in most countries have specific guidelines for testing drug candidates for genotoxicity as part of their safety evaluation [21]. Based on the rapid development of new nanoproducts, genotoxicity data on MNPs are required for the translation of these nanoproducts from research laboratories to the clinical market. The physicochemical and biological properties of nanomaterials are significantly different than their macroscopic counterparts. Decreasing the size of materials to the nanoscale, extraordinary increases the reactivity and subsequently interaction of NMs with the biological entities. Moreover, NMs are able to pass through the biological barriers and exert their beneficial or converse effects [22]. Hence the current safety assessment protocol applicable for materials in the bulk state must be customized to be applicable for NMs. There are some critical questions about the suitability of the current regulatory testing regime for NMs, the need for development of new and customized assessing methods, and the combination of different assessing method [23]. Figure 3 shows the interface of nature, nanotechnology, and genotoxicity. To the best of our knowledge, although the genotoxicity of non-biogenic MNPs prepared via conventional physical and chemical procedures has been discussed, no published work has comprehensively reviewed the genotoxicity of biologically synthesized MNPs. Therefore, the aim of the current study was to conduct a global systematic review evaluating the genotoxicity of biogenic MNPs in experimental investigations that were reported in the literature and involved in vitro and in vivo models.

## 2. Methods

### 2.1. Search Strategy

The genotoxicity of biologically synthesized MNPs was evaluated in a systematic review carried out by screening literature published online in the following databases; Cochrane, Web of Science, PubMed, Scopus, Science Direct, ProQuest, and EBSCO. The language was restricted to English in the literature search, and the published papers were searched from inception to June 30, 2018. The literature was searched by using different combinations of several keywords: ʻsynthesisʼ, ʻbiosynthesisʼ, ʻbiofabricationʼ, ʻgreenʼ, ʻbioreductionʼ, ʻmyco*ʼ, ʻbiogenicʼ, ʻPlant*ʼ, ʻphyto*ʼ, ʻherbal*ʼ, ʻfungus mediatedʼ, ʻbacterial mediatedʼ; ʻnanoparticle*ʼ, ʻcolloidalʼ, ʻnanomaterial*ʼ; and ʻgenotoxicityʼ, ʻgenotoxicʼ, ʻDNA fragmentationʼ, ʻDNA ladderʼ, ʻDNA damageʼ.

### 2.2. Inclusion Criteria

The scientific papers with the following characteristics were included; (1) original papers, (2) papers contain sufficient information, (3) papers obtained from the aforementioned key search, (4) published and/or in press papers, (5) English language papers, and (6) the papers that assessed the genotoxicity of biogenic MNPs through in vitro and/or in vivo models.

### 2.3. Exclusion Criteria

The papers with the following characteristics were excluded; (1) duplicated papers, (2) letters to the editor, (3) nonrelated papers, (4) case reports, (5) review papers, (6) congress posters, (7) editorials, (8) papers written in any language except English, (9) papers without full text, and (10) the papers that investigated the genotoxicity of chemical and/or physical-mediated fabricated MNPs.

### 2.4. Data Collection

The articles were reviewed in detail by two independent reviewers to assess their eligibility for inclusion. The data extraction form consisted of the first author, publication year, biological source, NP type, NP size (nm), NP morphology, in vitro model, in vivo model, NP dose, exposure time, genotoxicity assay, major genotoxicity comments, and genotoxicity (yes or no) (Tables S1 and S2).

## 3. Results

**Figure 4:**
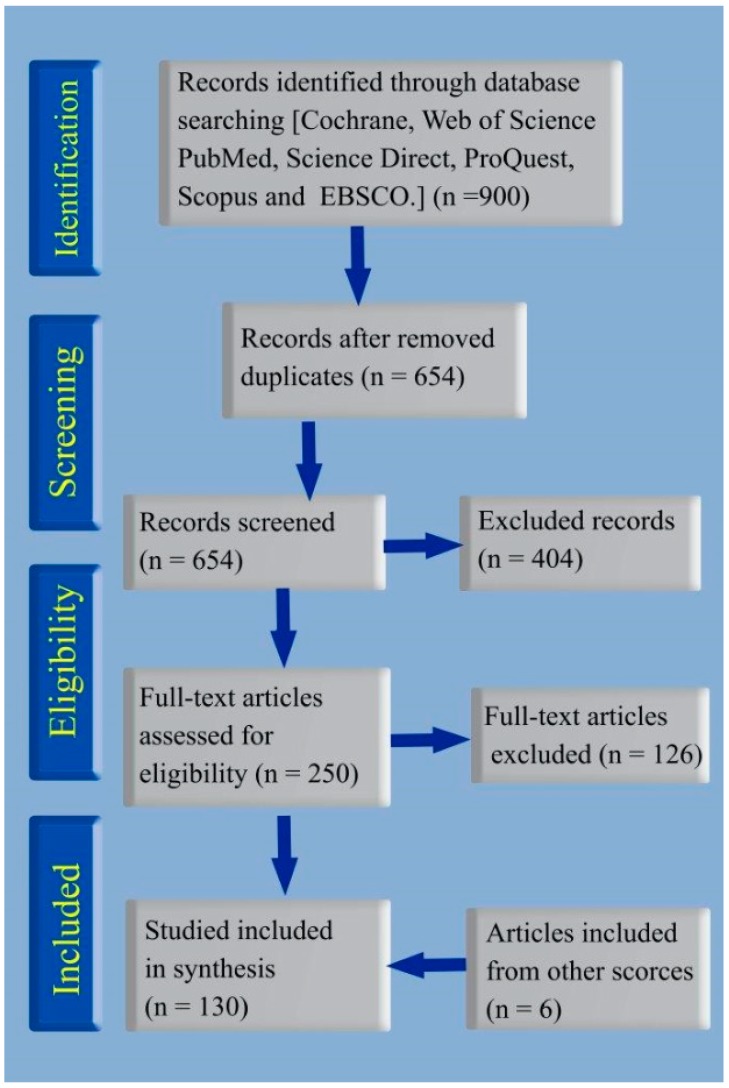
Flowchart describing the study design process.

Figure 4 shows the study design 1profile. The initial database search recovered 900 articles, of which 246 duplicates were removed. After screening the 654 articles, 404 articles were excluded. Of the remaining 250 articles, 126 articles were excluded. Searching other sources identified 6 additional articles. Ultimately, 130 articles were included in the current systematic review. Notably, considerable heterogeneity was found across all studies due to the different natural sources applied for the preparation of MNPs and the different types of MNPs, as well as the different genotoxicity analysis methods. The 130 articles included 122 in vitro studies and 8 in vivo studies and applied 153 different genotoxicity tests: DNA fragmentation assays (*n =* 58), the comet assay (*n =* 40), gene expression methods (*n =* 19), chromosomal aberration analysis (*n =* 9), cell cycle analysis (*n =* 8), the TUNEL assay (*n =* 7), micronucleus assays (*n =* 5), the Ames test (*n =* 2), and other tests (*n =* 5). The in vitro genotoxicity evaluations were carried out with different types of cancerous (*n =* 35) and normal (*n =* 9) cells, bacteria (*n =* 10), plants (*n =* 5), plasmids (*n =* 2), fungi (*n =* 1), parasites (*n =* 1), and calf thymus (*n =* 1). Most of the genotoxicity studies were carried out on MCF-7 (*n =* 25) and MDA-MB-231 (*n =* 9) cancer cell lines, and normal human lymphocytes were the major normal cell lines used (*n =* 11). Most of the genotoxic studies involving bacteria and plants were carried out with *Escherichia coli* (*n =* 5) and *Allium cepa* (*n =* 7). The in vivo genotoxicity evaluations were carried out on fish (*n =* 3), mice (*n =* 2), silkworms (*n =* 1), parasites (*n =* 1), yeast (*n =* 1), and marine fleas (*n =* 1). Phytosynthesis was the general approach to NP preparation in all studies (*n =* 88), and fungi were the second most common resource applied for NP synthesis (*n =* 18). Bacteria and algae were used to biosynthesize NPs in 16 and 9 studies, respectively. A total of 141 biogenic NPs, including Ag, Au, ZnO, TiO_2_, Se, Cu, Pt, Zn, Ag-Au, CdS, Fe_3_O_4_, Tb_2_O_3_, and Si-Ag NPs, were prepared in the 130 included articles. Notably, AgNPs, prepared in 68.79% (*n =* 97) of studies, and AuNPs, prepared in 12.76% (*n =* 18) of studies, were the two most predominant biogenic MNPs fabricated in the 130 included articles. Different morphologies of biogenic MNPs were reported: spherical (*n =* 76), mostly spherical (*n =* 19), spherical and other shapes (*n =* 18), and other shapes (no spherical) (*n =* 9). Eight studies reported no morphological data. The studies also reported different size distributions of NPs: ≤ 25 nm (*n =* 66), 25–50 nm (*n =* 46), 50–100 nm (*n =* 22), and 100 < nm (*n =* 5). Notably, 80.57% of studies synthesized biogenic MNPs under 50 nm. Table S1 provides data on the in vitro genotoxic effects of biogenic NPs for each biological source used to synthesize MNPs and Table S2 shows the in vivo genotoxicity outcomes. Although several studies reported no genotoxicity [24,25,26,27,28,29,30,31,32] or antigenotoxic activity [24,33,34,35,36] of biogenic MNPs, most of the studies reported genotoxic effects at specific concentrations and with a dose or time dependence.

## 4. Discussion

### 4.1. A Mechanistic Approach to Nanogenotoxicity

**Figure 5:**
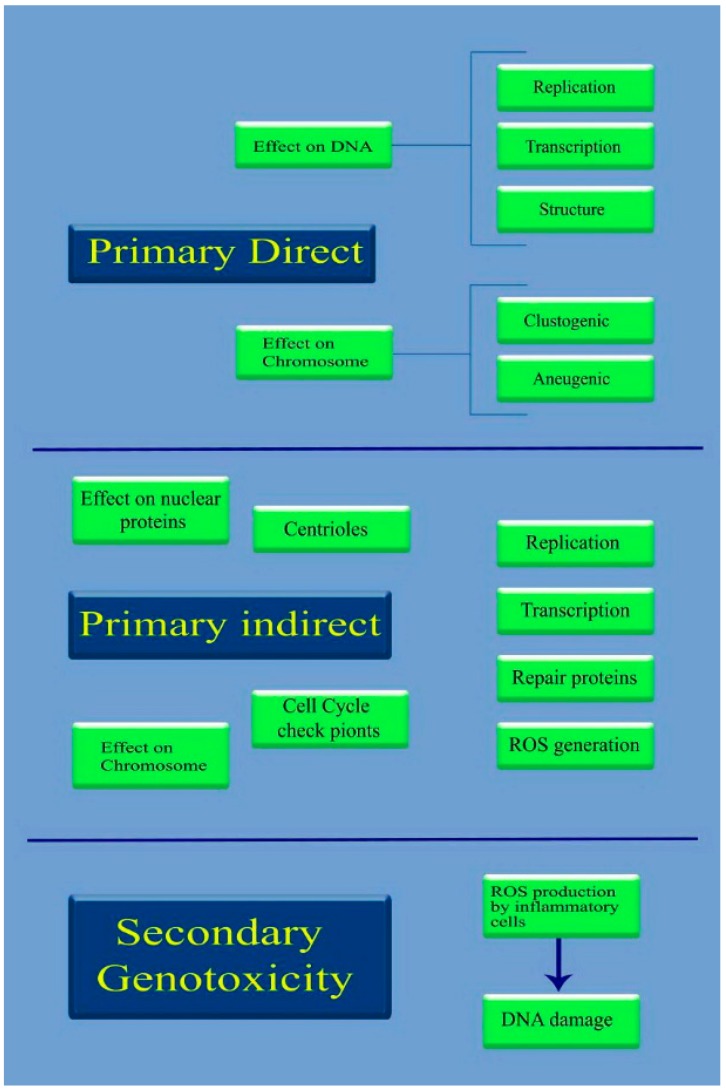
The genotoxicity pathways of nanomaterials. The primary effects can be categorized to primary direct (influencing on chromosome and/or DNA) and primary indirect (influencing on nuclear proteins and chromosomes). Secondary genotoxicity is mainly based on NPs-medicated oxidative stress.

**Figure 6:**
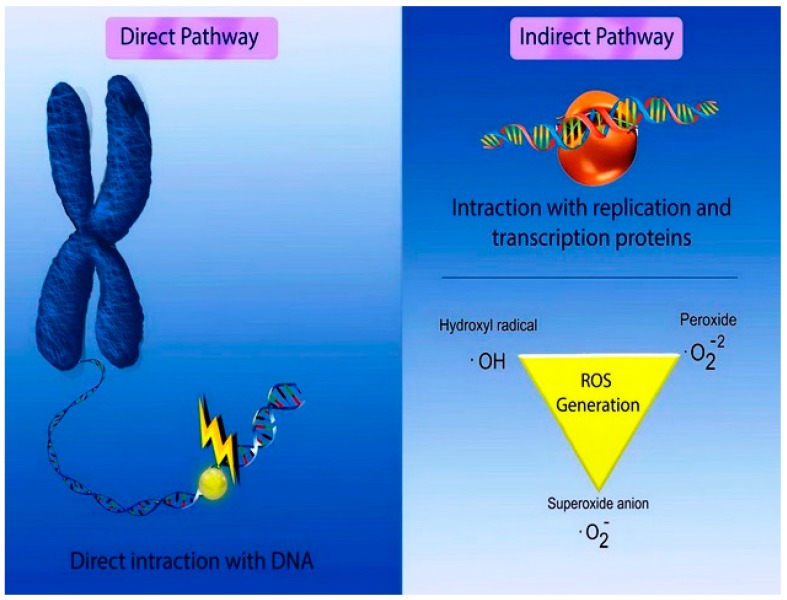
The genotoxic mechanisms of NPs. The direct pathway is based on the direct interaction of NPs with genomic materials, while the indirect pathway is through NPs-mediated generation of ROS such as hydroxyl, peroxide, and superoxide radicals.

The rapid development of nanoproducts in recent years has created significant concerns about their safety. Nanotoxicity as a new branch of toxicity can be described as the study of the adverse effects of nanomaterials on living organisms and ecosystems [37]. The potential of NPs to contaminate the environment and cause undesirable effects on human life should be clearly addressed before these nanoproducts reach the clinical market. Genotoxicity is a vital aspect of studying the damage to genetic information within a cell, such as DNA strand breakages, chromosomal fragmentation, point mutations, and alterations of gene expression profiles [38]. Nanogenotoxicity can be classified as primary or secondary. Primary NP-induced genotoxicity can be attributed to the direct or indirect interaction of NPs with genetic material. During direct primary genotoxicity, NPs directly interact with chromosomes during interphase and may bind with DNA molecules and prevent DNA replication or transcription. In addition, NPs could interact with chromosomes during mitosis, causing chromosomal breakage (clastogenic effect) or loss (aneugenic effect) mechanically or by chemical binding. During indirect primary genotoxicity, NP-mediated ROS generation and the release of toxic ions from soluble NPs are indirect interactions that can interfere with proteins essential for DNA replication, transcription, or repair as well as the mitotic spindle apparatus, centrioles or their associated proteins; these interactions can inactivate the proteins by structural modifications [39,40]. Figure 5 illustrates the direct and indirect primary genotoxicity pathways. ROS may cause purine- and pyrimidine-derived oxidized base lesions, which lead to mispairing in replication; consequently, destructive mutations may occur [41]. In addition, the protein kinases responsible for the regulation of cell cycle events and cell division may be inactivated by NPs, leading to a disturbance of cell cycle checkpoint functions [42]. Moreover, the secondary genotoxicity mechanism of NPs is attributed to the excessive generation of ROS by activated phagocytes, including neutrophils and macrophages, through a chronic in vivo inflammatory response [39,40]. Figure 6 summarizes the genotoxicity mechanisms of NPs. The genotoxicity of NPs may be affected by the NP properties, including the composition, size, shape, surface properties, physicochemical specifications (pH, temperature, etc.), solubility, and other factors such as the NP dose, exposure time, cell type used, and treatment regime [39,43,44]. The composition of NPs is the main driving force of the potential genotoxicity, for instance CdSe NPs are strongly toxic, regardless of their size, shape, and exposure route. Size is another influencing factor of genotoxicity which directly affect the surface to volume ratio, reactivity, solubility, and the exposure extent of the NPs with biological entities. Decreasing the size of NPs from the micro to nanoscale significantly increases the surface to volume ratio and the surface atoms. The increased reactivity results in the higher interaction and the interference with the biological entities which may induce higher ROS production. Moreover, the size and the shape of NPs impact the potential of genotoxicity, in the case of dissolved metallic ions-induced genotoxicity. The surface composition is also a critical parameter which determines types and the extent of the NP–biological system interaction, the dissolution rate of metallic ions, and the bio distribution of NPs [45]. Primary genotoxicity is usually evaluated with in vitro models, while secondary genotoxicity is investigated with in vivo models [39]. This study systematically reviews the genotoxic potential of biogenic MNPs determined with in vitro and in vivo models using documented data from a literature review.

### 4.2. In Vitro Genotoxicity Studies of Biogenic MNPs

#### 4.2.1. Genotoxicity Studies of Biogenic MNPs against Cancerous and Normal Cell Lines

There are various types of genotoxicity assessment tests available to evaluate the potential genotoxicity of NPs including: in vitro micronucleus assay (OECD TG 487), DNA assay by agarose gel electrophoresis, single cell gel electrophoresis assay (SCGE) or Comet assay, the cytokinesis-block micronucleus cytome (CBMN cyt) assay, Ames test (Bacterial Reversion Mutation Test) (OECD TG 471), 8-hydroxydeoxyguanosine DNA adducts detection, hypoxanthine–guanine hosphoribosyltransferase (HPRT) forward mutation assay, and g-H2AX staining [46,47,48,49]. In vitro genotoxicity assessments of MNPs have been performed against different cancerous and normal cell lines, including Y79 (human retinoblastoma), DLA (Dalton’s lymphoma), EAC (Ehrlich’s ascites carcinoma), MDA-MB-231 (human breast adenocarcinoma), MCF-7 (human breast adenocarcinoma), A549 (human lung adenocarcinoma), A375 (human malignant melanoma), AMJ-13 (human invasive ductal carcinoma), HeLa (human cervical cancer), B16-F10 (mouse melanoma), K562 (human leukemic), HT29 (human colorectal adenocarcinoma), HepG2 (human hepatocellular carcinoma), HCT-15 (human Dukes’ type C, colorectal adenocarcinoma), U87 (human glioblastoma), PC-3 (human prostate carcinoma), Caov-4 (human ovarian adenocarcinoma), KB (human carcinoma), MG-63 (human osteosarcoma), Saos-2 (human osteosarcoma), HT-29 (human colorectal adenocarcinoma), COLO 205 (human Dukes’ type D, colorectal adenocarcinoma), NCI-H460 (human nonsmall cell lung carcinoma), WEHI-3 (mouse leukemia), LNCap-FGC (human prostate carcinoma), MDA-MB (human adenocarcinoma mammary gland), MOLT-4 (human acute lymphoblastic leukemia), T47D (human ductal carcinoma), MCF10-A (human breast epithelial cell), Hep3B (human hepatocellular carcinoma), Hep2 (human carcinoma), HCT116 (human colorectal carcinoma), HL-60 (acute promyelocytic leukemia), SiHa (human cervical cancer cell), A431 (human epithelial carcinoma), RAW264.7 (mouse macrophage), ARPE-19 (human retinal pigment epithelium cell), HaCaT (human keratinocyte), normal human lymphocyte, PBMC (peripheral blood mononuclear cell), HEK293 (human embryonic kidney cell), 3T3 (mouse embryo), and V79 (hamster lung fibroblast). Maity et al., 2018, prepared AgNPs with a size of 3–15 nm by using an ethanolic extract of *Calotropis gigantea* latex. At 5.6 μg/mL, the biosynthesized AgNPs induced DNA fragmentation, cell cycle arrest at the G2/M phase, upregulation of Bax and caspase-3 and downregulation of Bcl-2 [50]. A similar DNA fragmentation result was reported by Reya et al. 2018, who evaluated the DNA damage of algal-based AgNPs (21–34 nm) at 10–50 μg/mL against the Y79 cell line [51]. Moteriya et al. 2018 investigated the genotoxicity of phytosynthesized AgNPs with an average size of 8 nm against normal human lymphocytes. No genotoxicity was found up to 50 µg, but fragmented DNA was observed at 200 µg [52]. Likewise, Moteriya et al. 2017 reported dose-dependent genotoxicity of *Caesalpinia pulcherrima* flower-synthesized AgNPs from 2 to 200 µg/mL in normal human lymphocytes. DNA fragmentation and chromatid gaps in chromosomes were found at 200 µg/mL, and the incidence of these effects were decreased at lower AgNP concentrations [53]. AgNPs with an average size of 49.98 nm synthesized via a fungus-mediated method caused DNA damage in NCI-H460 cells in a time-dependent manner at 2–20 µg [54]. Remarkably, several studies reported the nongenotoxicity of biologically synthesized MNPs. For instance, phytosynthesized CdSNPs with a size of 2–8 nm caused no DNA damage in normal human lymphocytes at 0.01 µg/mL as evaluated by using a comet assay, while exposure of normal human lymphocytes to pure commercially procured CdS resulted in extensive DNA damage [26]. Similarly, Das et al. 2017 reported no genotoxic influence of phytosynthesized AuNPs on normal human lymphocytes [28]. Phytosynthesized AuNPs at 6–15 µg/mL were also determined to be nongenotoxic in HaCaT cells using a comet assay [29].

#### 4.2.2. Genotoxicity Studies of Biogenic MNPs against Microbes and Parasites

The genotoxic influences of biogenic MNPs have been also evaluated by using different bacteria, including *Salmonella typhimurium*, *E. coli*, *Listeria monocytogenes*, *Salmonella typhi*, *Salmonella enterica*, *Pseudomonas aeruginosa*, *Bacillus cereus*, *Helicobacter pylori*, *Helicobacter felis*, and *Staphylococcus aureus*; two plasmids (pZPY112 and pBR322); one fungus (Aspergillus fumigatus); and one parasite (Leishmania donovani). Lv et al. 2018 reported that at 100 µg/mL, CuNPs with a size range of 6 to 20 nm synthesized via a bacterially mediated method induced DNA fragmentation in E. coli [55]. In addition, at 10 mg/mL, AgNPs (10–60 nm) synthesized via a plant-mediated method caused DNA fragmentation in *S. aureus* [56]. Similarly, at 1 µg/mL, phytosynthesized AgNPs with an average size of 20 nm resulted in fragmented DNA in *H. pylori* and *H. felis* [57]. In contrast, at concentrations of 1–3 mM, phytosynthesized AgNPs with sizes of 5–50 nm did not show any genotoxic effects against *B. cereus*, *S. aureus*, *L. monocytogenes*, *S. typhi*, or *S. enterica* [31]. Moreover, at 25 µg/mL, AgNPs synthesized by a bacterially mediated method caused DNA fragmentation in E. coli; however, no significant DNA damage was found against *S. typhimurium*, *P. aeruginosa*, or *S. aureus* at the same concentration [58]. The genotoxicity of TiO_2_ with an average size of 69 nm was investigated in the pBR322 plasmid. No genotoxicity was found at NP concentrations up to 250 µg/mL, but DNA deformations were found at 500 µg/mL [59]. Similarly, at 0.51 µg, a significant genotoxic effect of AgNPs with a size of 5 to 40 nm synthesized via a fungus-mediated method was reported for the pZPY112 plasmid [60]. In contrast, at concentrations of 6.25 to 25 µg/mL, phytosynthesized AgNPs with a size of 10 to 15 nm did not cause DNA damage in the pBR322 plasmid [27]. Notably, Neveen et al. 2013 reported that at 15 µg/mL, AgNPs with a size of 20 to 140 nm synthesized via an Aspergillus terreus-mediated method were genotoxic against A. fumigatus according to the comet assay [61]. Additionally, at 50 µM, phytosynthesized AgNPs with an average size of 35 nm did not cause DNA damage in *L. donovani* [30].

#### 4.2.3. Genotoxicity Studies of Biogenic MNPs against Plants

The genotoxicity of biogenic MNPs has been evaluated against the following plants; *Triticum aestivum* L., *Drimia indica*, *A. cepa*, *Lathyrus sativus* L., and *Brassica rapa*. Daphedar et al. 2018 investigated the genotoxic effect of ZnNPs with a size of 10–85 nm on the mitotic chromosomes of root tip meristematic cells in *D. indica*. The biogenic ZnNPs showed a mitodispersive effect on cell division and induced chromosomal abnormalities in a dose- and duration-dependent manner [62]. In addition, Panda et al. 2017 reported that phytosynthesized ZnONPs with an average size of 48.6 nm significantly induced DNA damage in a dose-dependent manner in *L. sativus* L., with maximum genotoxicity observed at 100 mg/L [63]. Abdelsalam et al. 2018 investigated the genotoxicity of AgNPs synthesized by an algally-mediated method at concentrations of 10 to 50 ppm on *T. aestivum* L. root tip cells. The AgNPs produced various types of chromosomal aberrations, such as an incorrect orientation at metaphase, chromosomal breakage, metaphasic plate distortion, spindle dysfunction, and stickiness [64]. Additionally, a study investigated the genotoxicity of AgNPs (average size of 20 nm) at concentrations of 5 to 20 µg/mL in mitotic and meiotic cells of *A*. *cepa*. Various chromosomal aberrations were observed even at low concentrations of AgNPs [65]. Similarly, AgNPs with sizes of 2 to 11 nm synthesized by a bacterially mediated method were studied for their genotoxic effect at concentrations of 1 to 10 mg/mL on *B. rapa*. ssp. *rapa* L. The AgNPs produced extensive DNA damage and altered the expression of genes involved in a variety of metabolic pathways as well as the inhibition of chlorophyll and anthocyanin biosynthesis and an overproduction of ROS [66].

### 4.3. In Vivo Genotoxicity Studies of Biogenic MNPs

In vivo investigations have been carried out on *Saccharomyces cerevisiae* (yeast), *Bombyx mori* (silkworm) larvae, *Cyprinus carpio* (fish) blood, *Poecilia reticulata* (fish) larvae, *Ceriodaphnia cornuta* (marine flea) adults, *Danio rerio* (zebrafish), *Leishmania major* (parasite) promastigotes, and mice bearing DAL tumor cells. Biogenic AgNPs at concentrations of 50 and 100 µg/mL caused significant DNA damage in *S. cerevisiae*, while insignificant DNA damage was observed at 12.5 and 25 µg/mL [67]. In addition, at 100 ppm, biogenic AgNPs induced significant DNA fragmentation in larvae of the mulberry silkworm *B. mori* [68]. AgNPs at 40 µg/mL led to remarkable DNA damage in adults of the microcrustacean *C*. *cornuta*, whereas in larvae of *P. reticulata*, 20 µg/mL AgNPs led to DNA damage [69]. Notably, AgNPs produced micronuclei and nuclear abnormalities such as blebbed nuclei, lobed nuclei, and notched nuclei in peripheral blood cells of zebrafish (*D. rerio*), indicating genotoxicity [70]. Moreover, phytosynthesized AgNPs fragmented DNA into approximately 100–150 bp pieces in DAL-induced tumor cells in mice bearing the DAL tumor model, suggesting mitochondrially mediated apoptosis in DAL cell lines [71].

### 4.4. Antigenotoxicity Studies of Biogenic MNPs

Sarac et al. 2018 investigated the antimutagenic activity of biogenic AgNPs at 250, 100 and 50 µg/plate in *S. typhimurium* TA98 and TA100 against 4-nitro-o-phenylenediamine (4-NPD: 3 µg/plate) and sodium azide (NaN_3_: 8 µg/plate) using the Ames test. The AgNPs had an effective antimutagenic effect at all tested concentrations on TA98 cells and at 250 and 100 µg/plate on TA100 cells [24]. Another study investigated antigenotoxic effect of TiO_2_NPs biosynthesized from *Turbinaria conoidesv* by an algally-mediated method. Genotoxicity studies were conducted for screening of Mutagenicity using Ames test, to prove this as an antigenotoxicant. TiO_2_NPs biosynthesized were evaluated for antigenotoxicity against Mitomycin C-induced genetic toxicity in human peripheral lymphocytes. It is well known that administration of Mytomycin C causes increased cell damage, which in turn leads to increased chromosome aberration wherein co-administration of TiO_2_NPs along with Mytomycin C reduced the frequency of Chromosome aberration and Ames test. Thus it was inferred from the present study that the TiO_2_NPs biosynthesized using *Turbinaria conoides* can be used as an efficient and potent drug owing of its antigenotoxicity and antimutagenicity property [33]. Moreover, a study reported the antigenotoxic effect of green-synthesized AgNPs from *Ocimum sanctum* leaf extract against cyclophosphamide (CP)-induced genotoxicity in normal human lymphocytes. CP administration to human lymphocytes culture caused reduction in mitotic index (MI) and increase in chromosomal damages. The AgNPs at concentrations of 50 to 200 µg/mL reduced the chromosomal damage caused by CP and there was an increase in MI. The biological way of synthesizing AgNPs has advantages like cost effectiveness and ecofriendly. Also, the bio-synthesized AgNPs of *O. sanctum* leaf extract was found to be a powerful genoprotective agent [34]. An aqueous leaf extract of lemon plant was used as a precursor for synthesis of colloidal SeNPs. MTT assay as well single cell gel electrophoresis assay or comet assay revealed that application of phytosynthesized SeNPs at 0.01 μg/μL resulted in less cell death of normal human lymphocytes and prevented DNA damage when cells were exposed to UVB radiation. The fluorescent property of SeNPs can be used as diagnostic agent. Further, their anti DNA damaging property can be investigated as a chemotherapeutic agent in cancer therapy [36]. In a similar study, phytosynthesized SeNPs from *Terminalia arjuna* leaf extract prevented the genotoxic effect of arsenite on normal human lymphocytes. Studies on cell viability using MTT assay and DNA damage using comet assay revealed that synthesized SeNPs at 0.01 μg/μL showed a promising chemoprotective effect against arsenite-induced cell death and DNA damage, respectively [35].

## 5. Conclusions

The present study systematically reviewed laboratory studies that evaluated the genotoxicity of biogenic MNPs by using in vitro and in vivo models. It was shown that MNPs could be synthesized using different biological sources, including plants, microorganisms, and even algae, in various shapes and sizes. Although several studies reported the antigenotoxic influence of biogenic MNPs, most studies demonstrated their genotoxicity. Performing genotoxicity studies with MNPs to identify their potential damage is immensely important. In the past decade, a large number of papers have reported green synthesis methods for MNPs that utilize biological pathways as an ecofriendly approach. However, laboratory studies on the genotoxicity of biologically synthesized MNPs are still scarce and exhibit significant heterogeneity. Hence, comparing and drawing conclusions from genotoxicity data is quite difficult. However, the results of this study emphasized that the biosynthesis of MNPs using natural products may not prevent MNP genotoxicity. A better understanding of the genotoxicity mechanisms of biogenic MNPs would enable us to prevent potential damage. The underlying genotoxicity mechanism of chemically synthesized NP have been discussed in the other review paper and the current review provides a valuable summary of the genotoxicity information on biologically synthesized MNPs reported in the literature from inception to June 30, 2018. Further well-designed genotoxicity studies using the same protocol are required to clearly address not only the factors affecting the genotoxicity of biogenic MNPs but also the mechanisms of MNP genotoxicity, with a particular need for more in vivo studies.

## Supplementary Materials

Supplementary materials can be found at https://www.mdpi.com/1010-660X/55/8/439/s1. Table S1: In vitro genotoxicity studies of biologically synthesized MNPs. Table S2: In vivo genotoxicity studies of biologically synthesized MNPs.
