# Supplementary material for: A Systematic Review of the Genotoxicity and Antigenotoxicity of Biologically Synthesized Metallic Nanomaterials: Are Green Nanoparticles Safe Enough for Clinical Marketing?
Source: Medicina (Kaunas). 2019 Aug 5;55(8):439. doi: 10.3390/medicina55080439 (PMC6722661; doi:10.3390/medicina55080439)
Supplement: Supplementary file 1 [file medicina-55-00439-s001.pdf]

Table S1. *In vitro* genotoxicity studies of biologically synthesized MNPs.

Author/Year	Biological source	NP type, Size (nm)	Morphology	In vitro model[a]	Dose	Exposure time	Applied test	Major genotoxicity comments	Geno toxicity	Ref
Sarac et al. 2018	*Streptomyces griseorubens* AU2 (Bacterium)	Ag, 5-20 nm	Spherical	*Salmonella typhimurium*	50-250 μg/plate	24 h	Ames	No genotoxicity; strongest anti-mutagenic activity in *S. typhimurium* TA98 at 250 μg/plate.	No	[1]
Abdelsalam et al. 2018	*Corallina elongate* (Algae)	Ag, 7.5-25 nm	Spherical	*Triticum aestivum* L.	10-50 ppm	8, 16, and 24 h	Chromosomal aberration	AgNPs caused various types of chromosomal aberrations, such as incorrect orientation at metaphase, chromosomal breakage, metaphasic plate distortion, spindle dysfunction, and stickiness.	Yes	[2]
Remya et al. 2018	*Turbinaria ornate* (Algae)	Ag, 21-34 nm	Spherical	Y79	10-50 μg/mL	24 h	DNA fragmentation	AgNPs induced DNA fragmentation at all concentrations.	Yes	[3]
Raajshree et al. 2018	*Turbinaria conoides* (Algae)	ZnO, 70-120 nm	Spherical	DLA	50 μg/mL	24 h	DNA fragmentation	ZnONPs induced DNA fragmentation.	Yes	[4]
Maity et al. 2018	*Calotropis gigantean* (Plant)	Ag, 3-15 nm	Mostly spherical	EAC	5.6 μg/mL	24 h	DNA fragmentation, cell cycle analysis and western blot analysis	AgNPs induced DNA fragmentation, cell cycle arrest at the G2/M phase, upregulation of Bax and caspase-3 and downregulation of Bcl-2.	Yes	[5]
Lv et al. 2018	*Shewanella loihica* PV-4 (Bacterium)	Cu, 6-20 nm	Spherical	*Escherichia coli*	100 μg/mL	12 h	DNA fragmentation	CuNPs induced DNA fragmentation.	Yes	[6]
Şahin et al. 2018	*Punica granatum* (Plant)	Pt, Average: 20.12 nm	Cubical and spherical	MCF-7	25 and 100 μg/mL	48 h	Comet	Slight DNA damage was observed at 25 μg/mL, while significant DNA damage was observed at 100 μg/mL.	Yes (dose dependent)	[7]
Sulaiman et al. 2018	*Albizia adianthifolia* (Plant)	Iron oxide, 32-100 nm	Spherical	AMJ-13 and MCF-7	1.8 μg/mL for AMJ-13 and 7.7 μg/mL for MCF-7	16 h	DNA fragmentation and comet	Genotoxicity and DNA damage were confirmed by both the DNA fragmentation assay and comet assay.	Yes	[8]

Table S1. *In vitro* genotoxicity studies of biologically synthesized MNPs.

Moteriya et al. 2018	*Caesalpinia pulcherrima* (Plant)	Ag, Average: 8 nm	Spherical	Normal human lymphocytes	2-200 μg	No data	Comet	No genotoxicity was observed at up to 50 μg, but fragmented DNA was found at 200 μg.	Yes (dose dependent)	[9]
Koca et al. 2018	*Mentha aquatic* (Plant)	TiO_2, Average: 69 nm	Spherical	pBR322 plasmid	62.5-500 μg/mL	24 h	DNA fragmentation	No genotoxicity was observed at up to 250 μg/mL, but DNA deformation was found at 500 μg/mL.	Yes (dose dependent)	[10]
Daphedar et al. 2018	*Albizia saman* (Plant)	Zn, 10-85 nm	Spherical	*Drimia indica*	4–16 μg/mL	6-24 h	Mitotic index and chromosomal aberration	ZnNPs had a mitodispersive effect on cell division and induced chromosomal abnormalities in a dose- and duration-dependent manner.	Yes	[11]
Yekeen et al. 2017	Cocoa pod husk and cocoa bean (Plant)	Ag, 4-54.22 nm	Spherical	*Allium cepa*	0.01–100 μg/mL	24, 48 and 72 h	Mitotic index and chromosomal aberration	AgNPs had a mitodispersive effect on cell division and induced chromosomal abnormalities.	Yes	[12]
Syed et al. 2017	*Rhizophora mangle* (Plant)	Ag, 10-60 nm	Spherical	*Staphylococcus aureus*	10 mg/mL	30 min	DNA fragmentation	AgNPs created fragmented DNA.	Yes	[13]
Pandurangan et al. 2017	*Perilla frutescens* (Plant)	Ag, Average: ~23 nm	Spherical and hexagonal	HeLa	0.1 and 0.2 mg/mL	24 h	Fluorescent Microscopy with AO and EB staining probes	The appearance of a green nucleus in AgNPs-treated cancer cells confirmed the induction of apoptosis. Moreover, chromatin and cytoplasm were condensed in the treated cells.	Yes	[14]
Jha et al. 2017	*Citrus maxima* (Plant)	Ag, 2-50 nm	Spherical	B16-F10	10 μg/mL	48 h	DNA fragmentation	AgNPs caused clear fragmentation of genomic DNA.	Yes	[15]
Fierascu et al. 2017	*Melissa officinalis* L. (Plant)	Ag, Au and Ag-Au, Average: 13 nm for Au, 10 nm for Ag, and 100 nm for Ag-	Spherical, triangular, hexagonal, rhombic, etc.	*Allium cepa*	10-20%	48 h	Mitotic index and chromosomal aberration	AgNPs were found not active on nuclear DNA damage. The AuNPs appeared nucleoprotective, but were aggressive in generating clastogenic aberrations in *A. cepa* root meristematic cells.	No (Ag) Yes (Au)	[16]

Table S1. *In vitro* genotoxicity studies of biologically synthesized MNPs.

Verma et al. 2017	*S. aureus, B. thuringiensis* (gram-positive bacteria), and *E. coli, S. typhimurium* (gram-negative bacteria)	Au bimetallic Ag, 4.8-29.3 nm	Spherical	HCT116	50 and 250 µg/mL	24 and 48 h	Cell cycle analysis via propidium iodide staining of the nucleus	AgNPs biosynthesized from gram-negative strains showed higher cytotoxicity than AgNPs biosynthesized from gram-positive strains and induced greater oxidative stress, morphological changes, apoptosis, and cell cycle arrest in the G0/G1 phase.	Yes	[17]
Prasad et al. 2017	*Asparagus racemosus* (Plant)	CdS, 2-8 nm	Mostly spherical	Normal human lymphocytes	0.01 µg/µL	1 h	Comet	No DNA damage was observed.	No	[18]
Syed et al. 2017	*Turbinaria conoides* (Algae)	TiO_2, 60-100 nm	Irregular	*Salmonella typhimurium* viz., and normal human lymphocytes	0.312–5 mg/mL	48 h for bacterial samples and 3-6 h for human samples	Ames and chromosomal aberration	TiO_2NPs caused no DNA damage against *S. typhimurium* viz. Biosynthesized NPs had a geno-protective nature and nonmutagenic effect on normal human lymphocytes.	No	[19]
Saha et al. 2017	*Swertia chirata* (Plant)	Ag, Average: 20 nm	Mostly spherical	*Allium cepa*	5–20 µg/mL	4 h	Chromosomal aberration	Various chromosomal aberrations were observed even at low concentrations of AgNPs.	Yes	[20]
Panda et al. 2017	*Calotropis gigantea* L. (Plant)	ZnO, Average: 48.6 nm	Spherical to hexagonal	*Lathyrus sativus* L.	0–100 mg/L	15 h	Comet	ZnONPs induced significant DNA damage in a dose-dependent manner.	Yes (dose dependent)	[21]
Moteriya et al. 2017	*Caesalpinia pulcherrima* (Plant)	Ag, 2-22 nm Average: 12 nm	Spherical	Normal human	2–200 µg/mL	96 h	Comet and chromosomal aberration	DNA fragmentation and chromatid gaps in chromosomes were observed at 200 µg/mL, and these	Yes	[22]

Table S1. *In vitro* genotoxicity studies of biologically synthesized MNPs.

				lymphocytes				effects were less prominent at lower AgNP concentrations.	(dose dependent)	
Datkhile et al. 2017	*Nothapodytes foetida* (Plant)	Ag, No data	No data	K562	5-25 μg/mL	48 h	DNA fragmentation	AgNPs caused extensive double-strand breaks and DNA damage.	Yes	[23]
Guilger et al. 2017	*Trichoderma harzianum* (Fungus)	Ag, 20-30 nm	Spherical	*Allium cepa* for chromosomal aberration assay. 3T3, HeLa, HaCaT, V79 and A549 cells for comet assay.	$[0.15–3.16] \times 10^{12}$ NPs/mL for the chromosomal aberration assay and $[0.15-0.47] \times 10^{12}$ NPs/mL for the comet assay	24 h for the chromosomal aberration assay and 1 h for the comet assay	Comet and chromosomal aberration	AgNPs caused DNA damage in all of the mammalian cell lines tested as well as changes in the mitotic index and the alteration index due to chromosomal aberrations.	Yes	[24]
Barua et al. 2017	*Thuja occidentalis* (Plant)	Ag, 10-15 nm Average: 12.7 nm	Spherical	Super coiled DNA of pBR322 plasmid and calf thymus DNA	6.25–25 μg/mL	48 h	DNA fragmentation	No DNA strand scission was observed in the supercoiled DNA of pBR322 or calf thymus DNA.	No	[25]
Bhanumathi et al. 2017	*Syzygium cumini* (Plant)	Ag, 15-30 nm	Spherical	MCF-7 and MDA-MB-231	10–100 μg/mL	24 and 48 h	Western blot	AgNPs activated p53 and Bax by downregulating Bcl-2 expression.	Yes	[26]
Das et al. 2017	*Syzygium cumini* (Plant)	Au, Average: 15 nm	Heterogeneously shape	Normal human lymphocytes	No data	No data	Chromosomal aberration	No genotoxicity was found.	No	[27]
Baghbani-Arani et al. 2017	*Artemisia tournefortiana* Rchb (Plant)	Ag, Average: 22.89±14.82 nm	Spherical	HT29 and HEK293	61.38 μg/mL for HEK293 and 40.71 μg/mL for HT29	24 h	Quantitative real-time PCR	The Bax/Bcl-2 ratio was upregulated. In detail, an increase and decrease in the mRNA level of Bax and the Bcl-2 expression in cell lines were observed.	Yes	[28]

Table S1. *In vitro* genotoxicity studies of biologically synthesized MNPs.

Banerjee et al. 2017	*Mentha arvensis* (Plant)	Ag, 3-9 nm	Spherical	MCF-7 and MDA-MB-231	1.56–12.5 μg/mL	2-48 h	Western blot	Upregulation of PARP1, P53, P21, Bax and cleaved caspase-9 was observed in MCF-7 cells, whereas Bcl2 was downregulated. In MDA-MB-231 cells, the mutant P53 protein was downregulated, whereas PARP1, P53, P21, Bax, cleaved caspase-9, procaspase-3 and cleaved caspase-3 proteins were upregulated.	Yes	[29]
Khalid et al. 2017	a) *Dictyosphaerium* sp. strain HM1 (DHM1) b) *Dictyosphaerium* sp. strain HM2 (DHM2) c) *Pectinodesmus* sp. strain HM3 (PHM3) (Microalgae)	Ag, Average: a) 22.5 nm for DHM1-AgNPs; b) 47.5 nm for DHM2-AgNPs; c) 57.5 nm for PHM3-AgNPs	DHM1-AgNPs and DHM2-AgNPs: spherical; PHM3-AgNPs: ovoid shape	MCF-7 and HepG2	10–50 μg/mL	24 h	DNA fragmentation	AgNPs caused DNA cleavage and fragmentation.	Yes	[30]
Datkhile et al. 2017	*Nothapodytes foetida* (Plant)	Ag, 10-50 nm	Spherical	MCF-7, HeLa, MCF-7, HCT15 and K562	1–20 μg/mL	48 h	DNA fragmentation	AgNPs caused DNA damage, upregulation of P53 and caspase-3 and downregulation of Bcl2 genes.	Yes	[31]
Chandrakasan et al. 2017	*Xenorhabdus stockiae* KT835471 (Bacterium)	Ag and Au, Average: 14±6 nm for AgNPs and 14±5 nm for AuNPs	AgNPs: spherical; AuNPs: spherical, ovoid and triangular	A549	29.4 μg/mL for AgNPs and 49.8 μg/mL for AuNPs	24 h	DNA fragmentation	AgNPs and AuNPs induced apoptosis through ROS-mediated DNA damage.	Yes	[32]
Das Nelaturi et al. 2017	*Allamanda Cathartica* L. (Plant)	Ag, Average: 35 nm	Spherical	PBMC	20–100 μg/mL	4 h	DNA fragmentation	AgNPs caused DNA cleavage and fragmentation.	Yes	[33]

Table S1. *In vitro* genotoxicity studies of biologically synthesized MNPs.

Daphedar et al. 2017	*Albizia saman* (Jacq.) Merr. (Plant)	Ag, 55-83 nm	Spherical, triangular and irregular	*Drimia indica* (Roxb.) Jessop	25–100%	6-24 h	Chromosomal aberration	AgNPs created abnormalities in chromosomes such as a sticky metaphase, single bridge at anaphase, normal anaphase with micronuclei, anaphase with chromosome fragments, laggard anaphase, multipolarity at anaphase, disturbed metaphase, diagonal anaphase, and C-metaphase.	Yes	[34]
Şuţan et al. 2016	*Asplenium scolopendrium* L. (Plant)	Ag, No data	No data	*Allium cepa* L.	No data	6-24 h	Chromosomal aberration	The plant extract supplemented with AgNPs incurred a variable incidence of C-mitosis, anaphase bridges, and sticky chromosomes alongside vagrant chromosomes.	Yes	[35]
Mishra et al. 2016	*Hibiscus sabdariffa* (Plant)	Au, Average: 30 nm	Spherical	U87	1–2.5 ng/mL	24 h	DNA fragmentation	AuNPs caused DNA damage in a dose-dependent manner.	Yes (dose dependent)	[36]
Qi et al. 2016	*Magnetospirillum gryphiswaldense* MSR-1 (Bacterium)	Fe_3O_4, Average: 30 nm	Subspheroidal	ARPE-19	10–200 μg/mL	24 h	Comet	Biogenic Fe_3O_4 caused less DNA damage than chemically synthesized Fe_3O_4. However, both were found to be genotoxic and caused DNA damage.	Yes	[37]
Perde-Schrepler et al. 2016	*Cornus mas* (Plant)	Au, 2-24 nm Average: 12.079 ± 3.588 nm	Round and oval	HaCaT	6–15 μg/mL	24-48 h	Comet	AuNPs showed low toxicity, caused minimal ROS production and did not induce additional DNA lesions or an increase in inflammatory cytokine production.	No	[38]
Suganya et al. 2016	*Mimosa pudica* (Plant)	Au, Average: 12.5 nm	Spherical	MDA-MB-231 and MCF-7	4 μg/mL for MDA-MB-231 and 6 μg/mL for MCF-7	48 h	Comet	AuNPs caused significant damage to the DNA and lengthened the tails of condensed DNA compared to that observed in the control in both cell lines.	Yes	[39]

Table S1. *In vitro* genotoxicity studies of biologically synthesized MNPs.

Suganya et al. 2016	*Musa paradisiaca* (Plant)	Au, <50 nm	Spherical	MCF-7 and MDA-MB-231	2 μg/mL for MDA-MB-231 and 8 μg/mL for MCF-7	48 h	Comet	Increased length of the comet tail (DNA damage) induced by AuNPs in both cell lines.	Yes	[40]
Panda et al. 2016	*Mangifera indica* L. (Plant)	Ag, 14-44.6 nm	Spherical	*Lathyrus sativus* L.	1–100 mg/L	3 h	Chromosomal aberration (CA), micronucleus formation and comet	AgNPs fabricated under four different reaction conditions caused DNA damage and were genotoxic. Moreover, using polyvinyl polypyrrolidone in the green synthesis of AgNPs resulted in an attenuation of their genotoxicity.	Yes	[41]
Farah et al. 2016	*Adenium obesum* (Plant)	Ag, 10-30 nm	Mostly spherical	MCF-7	50–150 μg/mL	24 h	Comet	AgNPs were found to be toxic at low concentrations (IC_{50}=73 μg/mL), with enhanced intracellular levels of ROS resulting in DNA damage, apoptosis and autophagy.	Yes	[42]
Elshawy et al. 2016	*Penicillium aurantiogriseum* (Fungus)	Ag, Average: 12.7 nm	Spherical	MCF-7	0.44–14 μg/mL	24 h	DNA fragmentation	AgNPs resulted in DNA strand breakage.	Yes	[43]
Jang et al. 2016	*Lonicera hypoglauca* (Plant)	Ag, 4.99-25.83 nm	Spherical	MCF-7	500 μg/mL	48 h	Western blot	AgNPs upregulated the p53 tumor suppressor gene and the subsequent increases in the expression of pro-apoptotic Bax, caspase-3 and caspase-9. In addition, AgNPs downregulated the mRNA levels of anti-apoptotic Bcl-2 and curtailed the JAK/STAT signaling in MCF-7 cancer cells.	Yes	[44]
Prabhu et al. 2016	*Setaria verticillata* L (Plant)	Ag, Average: 12±4 nm	Spherical	MCF-7 and A549	32.5-1000 μg/mL	24 h	DNA fragmentation	AgNPs resulted in double-strand breaks and the formation of DNA ladders in agarose gel, which are characteristic of apoptosis.	Yes	[45]

Table S1. *In vitro* genotoxicity studies of biologically synthesized MNPs.

Prasannaraj et al. 2016	a) *Plumbago zeylanica* b) *Semecarpus anacardium* c)*Terminalia arjuna* (Plant)	Ag, a) 80–98, b) 60–95 c) 34–70 nm	Spherical and cuboid	HepG2 and PC3	1-100 μg/mL	48 h	DNA fragmentation	AgNPs resulted in DNA ladder formation and DNA damage.	Yes	[46]
Kayalvizhi et al. 2016	*Curculigo orchioides* (Plant)	Ag, 15-18 nm	Spherical	MDA-MB-231	10–100 μg/mL	48 h	DNA fragmentation	AgNPs resulted in DNA strand break.	Yes	[47]
Selvi et al. 2016	*Padina tetrastromatica* (Algae)	Ag, 40-50 nm	Predominantly round	MCF-7	50-200 μg/mL	24 h	DNA fragmentation	AgNPs did not cause any DNA fragmentation at 50 μg/mL and induced very little fragmentation at 100 μg/mL, and moderate apoptotic fragmentation at 200 μg/mL.	Yes (dose dependent)	[48]
Chandramohan et al. 2016	*Azadirachta indica* (Plant)	Ag, 30-50 nm	Mostly spherical	Peripheral erythrocytes of goldfish (*Carassius auratus*)	0-12 ppm	72 and 96 h	Comet and micronucleus assay	AgNPs caused no significant damage at doses below 12 ppm. However, after 72 and 96 h, AgNPs at 12 ppm resulted in nuclear abnormalities and damage to the nuclear membrane.	Yes (dose dependent)	[49]
Kajani et al. 2016	*Taxus baccata* (Plant)	Ag, 75.1 and 91.2 nm	Spherical	Caov-4	2.5 and 5 μg/mL	24 and 48 h	DNA fragmentation	AgNPs caused slight DNA fragmentation after 24 h at 2.5 μg/mL but obvious laddering patterns and double-strand breaks after 48 h at 5 μg/mL.	Yes (dose dependent)	[50]
Kalangi et al. 2016	*Anethum graveolens* (Plant)	Ag, Average: 35 nm	Mostly spherical	*Leishmania donovani*	50 μM	48 h	DNA fragmentation	AgNPs created no DNA breaks.	No	[51]
He et al. 2016	*Dimocarpus longan Lour.* (Plant)	Ag, 9-32 nm	Spherical	PC-3	10 μg/mL	24 h	Western blot	AgNPs inhibited prostatic cancer PC-3 cells and induced a	Yes	[52]

Table S1. *In vitro* genotoxicity studies of biologically synthesized MNPs.

								decrease in stat 3, bcl-2, and survivin expression and an increase in caspase-3 expression.		
Bhakya et al. 2016	*Helicteres isora* (Plant)	Ag, 16-95 nm Average: 25.55 nm	Mostly spherical and oval	KB	70 µg/mL	48 h	Comet	AgNPs caused DNA damage as fragmented DNA tails, olive tails, and tail length alterations.	Yes	[53]
Iram et al. 2016	*Fusarium oxysporum* (Fungus)	Tb_2O_3, Average: 10 nm	Mostly spherical	MG-63 and Saos-2	0.102 µg/mL	24 h	Nuclear morphology analysis by DAPI staining	Tb_2O_3NPs caused nuclear fragmentation associated with DNA damage, including typical tubular staining patterns and condensed nuclei.	Yes	[54]
Mata et al. 2016	*Abutilon indicum* (Plant)	Au, 1-20 nm	Spherical	HT-29	210 µg/mL	24 and 48 h	TUNEL, western blot, and cell cycle analysis	AuNPs caused negligible necrosis. In addition, AuNPs caused cell cycle arrest at the G1/S transition phase. Furthermore, the expression levels of active caspases (3, 8, 9) increased with an increasing AuNP concentration.	Yes	[55]
Azmath et al. 2016	*Colletotrichum* sp. (Fungus)	Ag, 20-50 nm	Spherical, nearly spherical, triangular and hexagonal	*Escherichia coli*	25-100 µg/mL	30 min	DNA fragmentation	AgNPs caused DNA deformation and damage.	Yes	[56]
Ashe et al. 2016	*Cucurbita maxima* (Plant)	Ag, Average: 76.10 ± 0.8 nm	Spherical	Saos-2	0.05-0.25 mM	12 h	Comet	A combination of AgNPs with glucose-derived glycation products caused DNA damage and cell death via apoptotic pathways, while glycated products alone induced cell death by necrosis.	Yes	[57]
Balashanmugam et al. 2016	*Cassia Roxburghii* (Plant)	Au, 25-35 nm	Spherical	HepG2	30 µg/mL	24 h	DNA fragmentation	AuNPs created fragmented DNA.	Yes	[58]

Table S1. *In vitro* genotoxicity studies of biologically synthesized MNPs.

Balaji et al. 2016	*Trichoderma viride* (Fungus)	Ag, 5-40 nm	No data	MCF-7	40 and 130 µg/mL	24 h	DNA fragmentation and western blot	AgNPs created fragmented DNA. Moreover, western blot analysis showed inhibition of Bcl-2 and activation of Bax.	Yes	[59]
Qayyum et al. 2016	*Caryota urens, Pongamia glabra, Hamelia patens, Thevetia peruviana, Calendula officinalis, Tectona grandis, Ficus petiolaris, Ficus busking, Juniper communis, Bauhinia purpurea* (Plant)	Ag, 1-60 nm	Spherical, quasi-spherical, triangular and pentagonal	pBR322 plasmid	50 and 250 µg/mL	12 h	DNA fragmentation	AgNPs led to mild or little plasmid damage at 50 µg/mL, while severe plasmid DNA damage was found at 250 µg/mL.	Yes (dose dependent)	[60]
Thiruvengadam et al. 2015	*Bacillus marisflavi* (Bacterium)	Ag, 2-11 nm Average: 8 nm	Mostly spherical	Turnip (*Brassica rapa* ssp. rapa)	1-10 mg/L	12 h	DNA fragmentation, comet, and TUNEL	AgNPs caused extensive DNA damage and altered the expression of genes involved in a variety of metabolic pathways as well as the inhibition of chlorophyll and anthocyanin biosynthesis and an overproduction of ROS.	Yes	[61]
Parandhaman et al. 2015	*Rhizopus oryzae* (Fungus)	Si-Ag nanocomposite, Average: 20±4.5 nm	Mostly spherical	Genomic and plasmid DNA of *Escherichia coli* and *Pseudomonas aeruginosa*	0.25-2 mg/mL	5 h	DNA fragmentation	Exposure to Si-Ag nanocomposites resulted in a decrease in genomic and plasmid DNA band intensity in the treated cells in comparison to the control cells, indicating DNA damage, in a dose-dependent manner.	Yes	[62]
Jeyaraj et al. 2015	*Podophyllum hexandrum* (Plant)	Ag and Au, Ag: 12-40 nm;	No data	MCF-7	100 and 200 µg/mL for Ag and	24 h	DNA	AgNPs and AuNPs caused the upregulation of Bax, BCl2, caspase-6, caspase-9, PARP, and p53; the	Yes	[63]

Table S1. *In vitro* genotoxicity studies of biologically synthesized MNPs.

		Au: 5-35 nm			200 and 400 μg/mL for Au		fragmentation and western blot	downregulation of Bcl-2 and DNA fragmentation.		
Mata et al. 2015	*Abutilon indicum* (Plant)	Ag, 5-25 nm	Spherical	COLO 205	4-12 μg/mL	24 h	TUNEL and cell cycle assay	AgNPs induced no significant necrosis up to 8 μg/mL but caused 4% necrosis at 12 μg/mL. In addition, AgNPs arrested the cell cycle at the G1/S transition stage.	Yes (dose dependent)	[64]
Zahir et al. 2015	*Euphorbia prostrate* (Plant)	Ag and TiO_2, Average: 12.82± 2.50 nm for AgNPs and 83.22± 1.50 nm for TiO_2NPs	AgNPs: Spherical; TiO_2NPs: circular and irregularly shaped	*Leishmania donovani*	12.5-50 μg/mL	24 h	DNA fragmentation and TUNEL	DNA breakage was not extensive after AgNP exposure. However, high-molecular-weight DNA fragments of ~700 bp were observed, indicating that the mode of cell death may be largely due to necrosis.	Yes	[65]
Manna et al. 2015	*Lentinus squarrosulus* (Mont.) Singer (Fungus)	Ag, Average: 2.78 ± 1.47 nm	Mostly spherical	*Escherichia coli*	40 μg/mL	8 h	Flow cytometry	AgNPs caused an increase in side scattering intensity, supporting the internalization of AgNPs inside bacterial cells.	Yes	[66]
Subbaiya et al. 2015	*Nocardia mediterranei*-5016 (Fungus)	Ag, Average: 49.98 nm	Rod-shaped	NCI-H460	2-20 μg	24 and 48 h	Comet	AgNPs caused DNA damage in a time-dependent manner.	Yes (time dependent)	[67]
Gandhiraj et al. 2015	*Momordica charantia* (Plant)	Ag, Average: 96.3 nm	Spherical	MCF-7	12-100 μg/mL	24 h	DNA fragmentation	AgNPs caused DNA damage, resulting in fragmented DNA.	Yes	[68]
Dwivedi et al. 2015	*Pseudomonas aeruginosa* strain JS-11(Bacterium)	Ag, 5–23 nm	Mostly spherical	MCF-7	0.5-10 μg/mL	24 h	Cell cycle analysis and real-time PCR	The genes BCl2, cyclin D1, DNAJA1, E2F transcription factor 1, GPX1 and HSPA4 were upregulated. Some genes from the DNA damage and repair pathway, including XRCC2 and DDB1, were	Yes	[69]

Table S1. *In vitro* genotoxicity studies of biologically synthesized MNPs.

								negatively downregulated. Moreover, cell cycle analysis revealed an increase in the subG1 peak with a concomitant reduction in the G1 phase.		
Baskar et al. 2015	*Vitex negundo* L. (Plant)	Ag, 10-20 nm	Mostly spherical	*Brassica rapa* ssp. *pekinensis*	100-500 μg/mL	10 days	DNA fragmentation	Concentration-dependent DNA damage was observed in AgNP-treated plants. AgNPs at 500 μg/mL induced ROS generation and DNA damage.	Yes (dose dependent)	[70]
Chung et al. 2015	*Eclipta prostrate* (Plant)	ZnO, Average: 29±1.3 nm	Triangular, radial, hexagonal, rod-shaped, and rectangular	HepG2	1-500 μg/mL	24 h	DNA fragmentation	ZnONPs caused DNA damage as fragmented DNA.	Yes	[71]
Baharara et al. 2015	*Achillea biebersteinii* (Plant)	Ag, 10-40 nm	Spherical and pentagonal	MCF-7	1–100 μg/mL	24 and 48 h	Gene expression by RT-PCR	AgNPs downregulated the anti-apoptotic genes of the Bcl-2 family and unregulated the pro-apoptotic members, such as Bax.	Yes	[72]
Ramar et al. 2015	*Solanum trilobatum* (Plant)	Ag, 12.50-41.90 nm	Spherical	MCF-7	5-50 μg/mL	24 h	Western blot	AgNPs downregulated Bcl-2 but upregulated the activation of caspase-3 and caspase-9.	Yes	[73]
Gurunathan et al. 2015	*Bacillus tequilensis* (Bacterium)	Ag, Average: 20 nm	Spherical	MDA-MB-231	0-25 μg/mL	24 h	TUNEL and western blot	AgNPs induced DNA damage and cellular apoptosis via activation of p53, p-Erk1/2, and caspase-3 signaling and downregulation of Bcl-2.	Yes	[74]
Gurunathan et al. 2015	*Artemisia princeps* (Plant)	Ag, Average: 20 nm	Spherical	*Helicobacter pylori*, *Helicobacter felis*	1 μg/mL	12 h	DNA fragmentation	AgNPs induced DNA fragmentation.	Yes	[75]

Table S1. *In vitro* genotoxicity studies of biologically synthesized MNPs.

Ismail et al. 2015	*Pleurotus ostreatus* (Fungus)	Ag, 13.1-24.1 nm; Average: 17.5 nm	Spherical	MCF-7 and HepG2	No data	48 h	DNA fragmentation	AgNPs induced DNA fragmentation and apoptosis in HepG2 and MCF-7 cells via suppression of Bcl-2 gene expression; upregulation of BAX; downregulation of Bcl2; and simulation of caspase, P53 and cytochrome c gene expression.	Yes	[76]
Namvar et al. 2015	*Sargassum muticum* (Algae)	ZnO, 10-15 nm	Hexagonal	WEHI-3	20-100 μg/mL	24-72 h	Western blot	ZnONPs caused a decrease in Bcl-2 expression and an increase in the level of Bax, suggesting disruption of mitochondrial membranes.	Yes	[77]
Parveen et al. 2015	*Cassia auriculata* (Plant)	Ag and Au, Average: 21 nm for Au and 20 nm for Ag	Spherical	A549, LNCap-FGC, and MDA-MB	10-30 μg/mL	24 h	DNA fragmentation	Ag and AuNPs caused DNA cleavage and exhibited genotoxicity in all cell lines.	Yes	[78]
Raman et al. 2015	*Rosa indica* (Plant)	Ag, 23.52-60.83 nm	Spherical	HCT-15	30 μg/mL	24 h	Western blot	AgNPs downregulated Bcl-2 and upregulated the activation of caspase-3 and caspase-9.	Yes	[79]
Krishnaraj et al. 2015	*Malva crispa* (Plant)	Ag, 5-50 nm	Spherical	*Bacillus cereus, Staphylococcus aureus, Listeria monocytogenes, Salmonella typhi,* and *Salmonella enterica*	1-3 mM	30 min-24 h	DNA fragmentation	AgNPs did not show any genotoxic effects against any of the tested bacterial strains.	No	[80]
Hullikere et al. 2015	*Tragia involucrate* (Plant)	Ag, Within 100 nm	Rod-shaped	MOLT-4	10-100 μg/mL	24-72 h	DNA diffusion assay	DNA diffusion slightly increased, indicating genotoxicity.	Yes	[81]

Table S1. *In vitro* genotoxicity studies of biologically synthesized MNPs.

Govindaraju et al. 2015	*Sargassum vulgare* (Algae)	Ag, Average: 10 nm	Spherical	HL60	2.84 μg/mL	48 h	DNA fragmentation	AgNPs induced DNA fragmentation.	Yes	[82]
Ortega et al. 2015	*Cryptococcus laurentii* (BNM 0525) (Fungus)	Ag, Average: 35±10 nm	No data	MCF7, T47D, and MCF10-A	5 μg/mL	12 h	Western blot	Caspase-9 was overexpressed, and caspase-3/7 activity was increased in MCF7 and T47D; in MCF10-A cells, caspase and Bcl-2 were maintained at constant levels.	Yes	[83]
Raman et al. 2015	*Pleurotus djamor* var. (Plant)	Ag, 5-50 nm	Spherical	PC3	10 and 40 μg/mL	24 h	Comet	Cells treated with 40 μg/mL AgNPs showed a higher tail DNA than cells treated with 10 μg/mL AgNPs and control cells.	Yes (dose dependent)	[84]
Vijaya et al. 2014	*Ocimum sanctum* (Plant)	Ag, Average: >100 nm	Spherical	Normal human lymphocytes	50-200 μg/mL	48 h	Chromosomal aberration	AgNPs reduced the chromosomal damages due to cyclophosphamide and showed an antigenotoxic activity.	No	[85]
Rajasekharreddy et al. 2014	*Sterculia foetida* L. (Plant)	Ag, 6.9±0.2 nm	Spherical	HeLa	16 μg/mL	24 h	DNA fragmentation	AgNPs created extensive double-strand breaks.	Yes	[86]
Krishnasamy et al. 2014	*Indigofera aspalathoides* (Plant)	Ag, No data	No data	Hep3B	194.65 μg/mL	24 and 48 h	DNA fragmentation	AgNPs induced nucleosomal DNA fragmentation.	Yes	[87]
Prasad et al. 2014	*Terminalia arjuna* (Plant)	Se, 10-80 nm	Spherical	Normal human lymphocytes	0.01 μg/μL	1 h	Comet	SeNPs prevented the manifestation of genotoxic effects in lymphocytes treated with arsenite.	No	[88]
Sarkar et al. 2014	*Alternaria alternate* (Fungus)	ZnO, 45-150 nm Average: 75±5 nm	Spherical, triangular and hexagonal	Normal human lymphocytes	125-1000 μg/mL	3 h	Comet	A significant increase in DNA fragmentation was induced at 1000 μg/mL.	Yes (dose dependent)	[89]
Kumar et al. 2014	*Paederia foetida* (Plant)	Ag, 2-20 nm Average: 8.9±3.6 nm	Spherical	Calf thymus and *Escherichia coli*	0-50 μg/mL	1 h for calf thymus and	DNA fragmentation	No genotoxicity was found.	No	[90]

Table S1. *In vitro* genotoxicity studies of biologically synthesized MNPs.

						24 h for E. *coli*				
El-Kassas et al. 2014	*Corallina officinalis* (Algae)	Au, Average: 14.57±1 nm	Spherical	MCF-7	0.75-6 µg/mL	48 h	DNA fragmentation	No DNA damage was found up to 1.5 µg/mL; however, significant DNA damage was observed at 3 and 6 µg/mL.	Yes (dose dependent)	[91]
Chowdhury et al. 2014	*Macrophomina phaseolina* (Fungus)	Ag, 5-40 nm; most were 16-20 nm	Spherical	pZPY112 plasmid	0.51-5.1 µg	2 h	DNA fragmentation	Genotoxicity was manifested as the degradation of plasmids, even at low concentrations.	Yes	[92]
Krishnaraj et al. 2014	*Acalypha indica* Linn (Plant)	Ag and Au, 20-30 nm	Spherical	MDA and MB-231	1-100 µg/mL	48 h	DNA fragmentation	Both AgNPs and AuNPs caused DNA damage and fragmentation.	Yes	[93]
Lima et al. 2014	*Fusarium oxysporum* (Fungus)	Ag, Average: 40.3±3.5 nm	Spherical	3T3, normal human lymphocytes and *Allium cepa*	0.5-10 µg/mL	1 h for 3T3 cells and human lymphocytes and 24 h for the *Allium cepa* assay	Comet and chromosomal aberration	AgNPs at 5 and 10 µg/mL had a genotoxic effect; however, at 0.5-1 µg/mL, no genotoxicity was observed.	Yes (dose dependent)	[94]
Singh et al. 2014	*Anabaena doliolum* (Bacterium)	Ag, 10-50 nm	Spherical	COLO 205	1-50 µg/mL	24 h	DNA fragmentation	DNA fragmentation increased significantly with an increasing AgNP concentration.	Yes (dose dependent)	[95]
Varun et al. 2014	*Argemone mexicana* (Plant)	Au, Average: 26±5 nm	Spherical	MCF-7	100 µg/mL	48 h	DNA fragmentation	AuNPs caused extensive double-stranded DNA breaks.	Yes	[96]

Table S1. *In vitro* genotoxicity studies of biologically synthesized MNPs.

Subbaiya et al. 2014	*Streptomyces olivaceus* sp-1392 (Bacterium)	Ag, Average: 200 nm	Spherical	NCI-H460	9.48 and 12.52 μg/mL	24 and 48 h	Comet	AgNPs caused DNA breakage and damage by increasing the amount of tail DNA, tail length, and olive tail moment.	Yes	[97]
Ashokkumar et al. 2014	*Cajanus cajan* (Plant)	Au, 9-41 nm	Spherical	HepG2	246 μg/mL	48 h	Comet	AuNPs caused DNA damage by increasing the amount of tail DNA, tail length, tail moment, and olive tail moment in HepG2 cells.	Yes	[98]
Jeyaraj et al. 2014	*Podophyllum hexandrum* L. (Plant)	Au, Average: 15 nm	Spherical and triangular	HeLa	20 μg/mL	24 h	Comet, western blot, and DNA fragmentation	AuNPs increased the amount of tail DNA, tail length, tail moment and olive tail moment in HeLa cells. A DNA ladder was formed in AuNP-treated cells. The level of Bcl-2 expression was reduced, and the level of Bax was increased.	Yes	[99]
Prasad et al. 2013	Lemon plant	Se, 60-80 nm	Spherical	Normal human lymphocyte s	0.01 μg/μL	1 min	Comet	SeNPs caused less cell death of lymphocytes and prevented DNA damage when cells were exposed to UVB radiation.	No	[100]
Rosarin et al. 2013	*Phyllanthus emblica* (Plant)	Ag, Average: 188 nm	Spherical and cubic	Hep2	20 μg/mL	24 h	DNA fragmentation	AgNPs caused DNA fragmentation.	Yes	[101]
Neveen et al. 2013	*Aspergillus terreus* (Fungus)	Ag, 20-140 nm	Spherical	*Aspergillus fumigatus*	15 μg/mL	36 h	Comet	AgNPs caused DNA damage and an increase in DNA tail length.	Yes	[102]
Mohanty et al. 2013	a) *Alstonia macrophylla* (Plant)<br>b) *Trichoderma* sp. (Fungus)	Ag, Average: a) 50 nm; b) 100 nm	Spherical	RAW264.7 macrophage s	5 and 10 ppm	12 h for the comet assay and 6 h for the micron ucleus assay	Comet and micronucleus assay	No DNA damage was observed at 5 ppm; however, significant micronuclei formation and DNA damage were observed at 10 ppm for both phytosynthesized and mycosynthesized AgNPs.	Yes (dose depen dent)	[103]

Table S1. *In vitro* genotoxicity studies of biologically synthesized MNPs.

Jeyaraj et al. 2013	*Sesbania grandiflora* L. (Plant)	Ag, Average: 22 nm	Spherical	MCF-7	0-50 μg/mL	24 and 48 h	Comet	AgNPs caused DNA breakage in the form of tail formation.	Yes	[104]
Jeyaraj et al. 2013	*Podophyllum hexandrum* L. (Plant)	Ag, 12-40 nm	Mostly spherical	HeLa	20 μg/mL	24 h	Comet, western blot, and DNA fragmentation	AgNPs increased the amount of tail DNA, tail length, tail moment and olive tail moment in HeLa cells. A DNA ladder was formed in AgNP-treated cells. The level of Bcl-2 expression was reduced, and the level of Bax was increased.	Yes	[105]
Prabhu et al. 2013	*Vitex negundo* L. (Plant)	Ag, 5-47 nm	Spherical	HCT15	20 and 100 μg/mL	48 h	Comet	AgNPs caused long tail formation and DNA damage.	Yes	[106]
Geetha et al. 2013	*Couroupita guianensis* (Plant)	Au, 7-48 nm	Spherical, triangular, tetragonal and pentagonal with irregular contours	HL-60	60-180 μg/mL for the comet assay and 0-150 μg/mL for the DNA fragmentation assay	48 h	Comet and DNA fragmentation	AuNPs caused long tail formation (DNA damage) and genotoxicity.	Yes	[107]
Govender et al. 2013	*Albizia adianthifolia* (Plant)	Ag, 4-35 nm	Mostly spherical	A549	43 μg/mL	6 h	Comet and western blot	Fragmentation of DNA was significantly induced by AgNPs. In addition, AgNPs increased the expression of p53, Bax and PARP-1.	Yes	[108]
Chunyan et al. 2013	a) Mint; b) Coffee; c) Ginger (Plant)	Ag, a) 5-10 nm; b) 30-40 nm; c) 5-10 nm and 30-40 nm	Mostly spherical	HeLa and HepG2	20 μg/mL	24 h	Cell cycle analysis	AgNPs caused DNA damage followed by cell cycle arrest in the G2/M stage and eventually cell death through apoptosis. This DNA damage was more significant for AgNPs synthesized by a mint-mediated method.	Yes	[109]
Gurunathan et al. 2013	*Ganoderma neo-japonicum* (Fungus)	Ag, 10-15 nm	Spherical	MDA-MB-231	6 μg/mL	24 h	DNA fragmentation and TUNEL	AgNPs induced cell death through ROS generation, caspase-3 activation, and DNA fragmentation.	Yes	[110]

Table S1. *In vitro* genotoxicity studies of biologically synthesized MNPs.

Gurunathan et al. 2013	*Bacillus funiculus* (Bacterium)	Ag, Average: 20 nm	Mostly spherical	MDA-MB-231	8.7 µg/mL	24 h	DNA fragmentation	AgNPs induced DNA fragmentation.	Yes	[111]
Wu et al. 2013	*Polyporus rhinoceros* (Fungus)	Se, Average: 50 nm	Spherical	A549	10 and 20 µM	24 h	TUNEL-DAPI costaining assay and cell cycle analysis	SeNPs induced G2/M phase arrest. In addition, DNA fragmentation and nuclear condensation were detected.	Yes	[112]
Gurunathan et al. 2013	*Escherichia fergusoni* (Bacterium)	Ag, 10-80 nm	Spherical	MCF-7	17.4 µg/mL	24 h	DNA fragmentation	AgNPs induced DNA fragmentation.	Yes	[113]
Tamboli et al. 2013	*Exiguobacterium* sp. KNU1 (Bacterium)	Ag, 5-50 nm	Spherical	*Salmonella typhimurium*, *Pseudomonas aeruginosa, Escherichia coli* and *Staphylococcus aureus*	25 µg/mL	4 h	DNA fragmentation	AgNPs revealed the fragmentation of DNA in the *E. coli* cells; however, no significant DNA damage was found in other bacteria.	Yes	[114]
Das et al. 2013	*Phytolacca decandra, Gelsemium sempervirens, Hydrastis canadensis* and *Thuja occidentalis* (Plant)	Ag, Average: approximately 100 nm	Mostly spherical	A375	80 and 160 µg/mL	24 h	Comet, cell cycle assay and DNA fragmentation	A DNA fragmentation study showed smear DNA in agarose gel, indicating DNA damage, while the comet assay did not show any fragmented DNA. AgNPs inhibited DNA synthesis and cell proliferation through G2/M cell cycle arrest.		[115]
Bhattacharyya et al. 2012	*Phytolacca decandra* (Plant)	Ag, Average: 91 nm	Spherical	A549	80 and 100 µg/mL	24 h	Comet and DNA fragmentation	AgNPs caused DNA fragmentation as well as an increase in DNA tail formation, indicating genotoxicity.	Yes	[116]
Mishra et al. 2012	*Azadirachta indica* (Neem) (Plant)	Ag, 2–18 nm	Spherical	SiHa	4, 8, 30, and 60 µg/mL	48 h	DNA fragmentation	AgNPs caused extensive double-strand breaks, thereby yielding a ladder-like appearance on agarose gel.	Yes	[117]

Table S1. *In vitro* genotoxicity studies of biologically synthesized MNPs.

Bendale et al. 2012	*Dolichos biflorous, Ocimum sanctum, Euphorbia neriifolia, Sesbania grandiflora, Piper betle, Calospropris procera, Asteracantha longifolia* (Plant)	Pt, Average: 137.5 nm	Cubic	A375	100 µg/mL	24, 48, and 72 h	DNA fragmentation	PtNPs induced DNA damage in a time-dependent manner.	Yes (time dependent)	[118]
Sarkar et al. 2011	*Alternaria alternate* (Fungus)	Ag, 20-45 nm Average: 28±4 nm	Spherical	Normal human lymphocytes	50-400 µg/mL	3 h	Comet	AgNPs caused significant DNA tail formation at 300 µg/mL; however, slight DNA damage was observed at lower concentrations.	Yes (dose dependent)	[119]
Satyavani et al. 2011	*Citrullus colocynthis* (Plant)	Ag, Average: 31 nm	Spherical	HEp-2	500 nM	6 h	DNA fragmentation	AgNPs caused extensive double-strand breaks.	Yes	[120]
Panda et al. 2011	*Pandanus odorifer* (Plant)	Ag, 24-55 nm Average: 37±11 nm	Mostly spherical	*Allium cepa* L.	5-80 µg/mL	12, 24 and 48 h	Comet and micronucleus assay	AgNPs induced DNA damage in a dose-dependent manner. DNA damage was significantly enhanced at doses ≥20 µg/mL.	Yes (dose dependent)	[121]
Singh et al. 2010	*Actinobacter* spp. (Bacterium)	TiO_2 and ZnO, Average: 5.5 nm for TiO_2 and 7 nm for ZnO	Spherical	A431	10^{-3}- 10^{-12} M	3 h	Comet	ZnONPs at concentrations up to 10^{-5} M caused DNA damage as a significant increase in the percentage of tail DNA. TiO_2 at concentrations up to 10^{-3} M did not cause a significant increase in percentage tail DNA.	Yes (dose dependent)	[122]

[a]**Cancer and normal cell Lines:** Y79 (human retinoblastoma), DLA (Dalton's lymphoma), EAC (Ehrlich's ascites carcinoma), MDA-MB-231 (human breast adenocarcinoma), MCF-7 (human breast adenocarcinoma), A549 (human lung adenocarcinoma), A375 (human malignant melanoma), AMJ-13 (human invasive ductal carcinoma), HeLa (human cervical cancer), B16-F10 (mouse melanoma), K562 (human leukemic), HT29 (human colorectal adenocarcinoma), HepG2 (human hepatocellular carcinoma), HCT-15 (human Dukes' type C, colorectal adenocarcinoma), U87 (human glioblastoma), PC-3 (human prostate carcinoma), Caov-4 (human

Table S1. *In vitro* genotoxicity studies of biologically synthesized MNPs.

ovarian adenocarcinoma), KB (human carcinoma), MG-63 (human osteosarcoma), Saos-2 (human osteosarcoma), HT-29 (human colorectal adenocarcinoma), COLO 205 (human Dukes' type D, colorectal adenocarcinoma), NCI-H460 (human nonsmall cell lung carcinoma), WEHI-3 (mouse leukemia), LNCap-FGC (human prostate carcinoma), MDA-MB (human adenocarcinoma mammary gland), MOLT-4 (human acute lymphoblastic leukemia), T47D (human ductal carcinoma), MCF10-A (human breast epithelial cell), Hep3B (human hepatocellular carcinoma), Hep2 (human carcinoma), HCT116 (human colorectal carcinoma), HL-60 (acute promyelocytic leukemia), SiHa (human cervical cancer cell), A431 (human epithelial carcinoma), RAW264.7 (mouse macrophage), ARPE-19 (human retinal pigment epithelium cell), HaCaT (human keratinocyte), normal human lymphocyte, PBMC (peripheral blood mononuclear cell), HEK293 (human embryonic kidney cell), 3T3 (mouse embryo), and V79 (hamster lung fibroblast).

Table S2. *In vivo* genotoxicity studies of biologically synthesized MNPs.

Author/Year	Biological source	NP type, Size (nm)	Morphology	*In vivo* model	Dose	Exposure time	Genotoxicity assay	Major genotoxicity comments	Genotoxicity (Yes or No)	Ref
Adiguzel et al. 2018	*Streptomyces* sp. AOA21 (Bacterium)	Ag, 35-60 nm	Spherical	*Saccharomyces cerevisiae*	12.5-100 μg/mL	3 h	Comet	AgNPs at 12.5 and 25 μg/mL led to insignificant DNA damage. However, AgNPs at 50 and 100 μg/mL resulted in significant DNA damage.	Yes (dose dependent)	[123]
Pandiarajan et al. 2018	*Morus alba* (Plant)	Ag, No data	No data	Larva of mulberry silkworm *Bombyx mori*	1-100 ppm	No data	DNA fragmentation, and Bm-actin amplification	A high mortality rate at 100 ppm and a moderate mortality rate at 10 ppm were observed during larval-pupal transition and pupal-adult transition. Significant DNA fragmentation was observed at 100 ppm. In addition, Bm-actin marker gene amplification revealed the null amplification at 10 and 100 ppm, respectively.	Yes (dose dependent)	[124]

Table S2. *In vivo* genotoxicity studies of biologically synthesized MNPs.

Gavade et al. 2017	*Ziziphus jujuba* (Plant)	Cu_xO/ZnO, Ag@Cu_xO/ZnO and Au@Cu_xO/ZnO (x= I and II), 15-40 nm	Hexagonal and irregular	*Cyprinus carpio* blood	No data	No data	Comet	NPs significantly induced genotoxicity even at low concentrations.	Yes	[125]
Ishwarya et al. 2017	*Cissus quadrangularis* (Plant)	Ag, No data	No data	Larvae of *Poecilia reticulata* fishes and adults of the microcrustacean *Ceriodaphnia cornuta*	10, 20 and 40 µg/mL	24 h	DNA fragmentation	AgNPs at 40 µg/mL led to remarkable DNA damage in *C. cornuta*, whereas in *P. reticulata*, 20 µg/mL AgNPs led to DNA damage.	Yes	[126]
Krishnaraj et al. 2016	*Malva crispa* Linn. (Plant)	Ag, 5-50 nm	Spherical	Zebrafish (*Danio rerio*)	23.7-331.8 µg/L	96 h	Micronuclei and nuclear abnormality test	AgNPs showed micronuclei and nuclear abnormalities such as blebbed nuclei, lobed nuclei, and notched nuclei in peripheral blood cells, indicating genotoxicity.	Yes	[127]
Beheshti et al. 2013	*Bacillus* sp. MSh-1 (Bacterium)	Se, 80-220 nm	Spherical	*Leishmania major* promastigotes	1-150 µg/mL	24 h	DNA fragmentation	SeNPs induced DNA fragmentation in a dose-dependent manner.	Yes (dose dependent)	[128]
Antony et al. 2013	*Ficus religiosa* (Plant)	Ag, 5-35 nm	Spherical	Dalton's ascites lymphoma (DAL) in a mouse model	25-100 µg/mL	11 days	DNA fragmentation	AgNPs caused DNA damage in DAL cells by initiating apoptosis.	Yes	[129]

Table S2. *In vivo* genotoxicity studies of biologically synthesized MNPs.

Sukirtha et al. 2011	*Areca catechu* Linn. (Plant)	Ag, Average: 80 nm	Spherical	Mice bearing DAL tumor cells	600-1000 μg	10 days	DNA fragmentation	AgNPs created fragmented DNA in DAL-induced tumor cells.	Yes	[130]

References

1. Sarac, N.; Baygar, T.; Ugur, A. In vitro mutagenic and anti-mutagenic properties of green synthesised silver nanoparticles. *IET Nanobiotechnol.* **2018**, *12*, 230–233. doi:10.1049/iet-nbt.2017.0016.
2. Abdelsalam, N.R.; Abdel-Megeed, A.; Ali, H.M.; Salem, M.Z.M.; Al-Hayali, M.F.A.; Elshikh, M.S. Genotoxicity effects of silver nanoparticles on wheat (*Triticum aestivum* L.) root tip cells. *Ecotoxicol. Environ. Saf.* **2018**, *155*, 76–85. doi:10.1016/j.ecoenv.2018.02.069.
3. Remya, R.R.; Rajasree, S.R.R.; Suman, T.Y.; Aranganathan, L.; Gayathri, S.; Gobalakrishnan, M.; Karthih, M.G. Laminarin based AgNPs using brown seaweed *Turbinaria ornata* and its induction of apoptosis in human retinoblastoma Y79 cancer cell lines. *Mater. Res. Express* **2018**, *5*. doi:10.1088/2053¨C1591/aab2d8.
4. Raajshree, K.R.; Durairaj, B. In vitro anticancer potential of biosynthesized zinc oxide nanoparticles from the seaweed *Turbinaria conoides*. *Asian J. Pharm. Clin. Res.* **2018**, *11*, 127–130. doi:10.22159/ajpcr.2018.v11i5.22224.
5. Maity, P.; Bepari, M.; Pradhan, A.; Baral, R.; Roy, S.; Maiti Choudhury, S. Synthesis and characterization of biogenic metal nanoparticles and its cytotoxicity and anti-neoplasticity through the induction of oxidative stress, mitochondrial dysfunction and apoptosis. *Colloids Surf. B Biointerfaces* **2018**, *161*, 111–120. doi:10.1016/j.colsurfb.2017.10.040.
6. Lv, Q.; Zhang, B.; Xing, X.; Zhao, Y.; Cai, R.; Wang, W.; Gu, Q. Biosynthesis of copper nanoparticles using *Shewanella loihica* PV-4 with antibacterial activity: Novel approach and mechanisms investigation. *J. Hazard. Mater.* **2018**, *347*, 141–149. doi:10.1016/j.jhazmat.2017.12.070.
7. Şahin, B.; Aygün, A.; Gündüz, H.; Şahin, K.; Demir, E.; Akocak, S.; Şen, F. Cytotoxic effects of platinum nanoparticles obtained from pomegranate extract by the green synthesis method on the MCF-7 cell line. *Colloids Surf. B Biointerfaces* **2018**, *163*, 119–124. doi:10.1016/j.colsurfb.2017.12.042.
8. Sulaiman, G.M.; Tawfeeq, A.T.; Naji, A.S. Biosynthesis, characterization of magnetic iron oxide nanoparticles and evaluations of the cytotoxicity and DNA damage of human breast carcinoma cell lines. *Artif. Cells Nanomed. Biotechnol.* **2018**, *46*, 1215–1229. doi:10.1080/21691401.2017.1366335.
9. Moteriya, P.; Chanda, S. Biosynthesis of silver nanoparticles formation from *Caesalpinia pulcherrima* stem metabolites and their broad spectrum biological activities. *J. Genet. Eng. Biotechnol.* **2018**, *16*, 105–113. doi:10.1016/j.jgeb.2017.12.003.
10. Koca, F.D.; Duman, F. Genotoxic and cytotoxic activity of green synthesized TiO_2 nanoparticles. *Appl. Nanosci.* **2018**. doi:10.1007/s13204-018-0712-1.
11. Daphedar, A.; Taranath, T.C. Green synthesis of zinc nanoparticles using leaf extract of *Albizia saman* (Jacq.) Merr. and their effect on root meristems of *Drimia indica* (Roxb.) Jessop. *Caryologia* **2018**, *71*, 93–102. doi:10.1080/00087114.2018.1437980.
12. Yekeen, T.A.; Azeez, M.A.; Lateef, A.; Asafa, T.B.; Oladipo, I.C.; Badmus, J.A.; Adejumo, S.A.; Ajibola, A.A. Cytogenotoxicity potentials of cocoa pod and bean-mediated green synthesized silver nanoparticles on *Allium cepa* cells. *Caryologia* **2017**, *70*, 366–377. doi:10.1080/00087114.2017.1370260.
13. Syed, B.; Bisht, N.; Bhat, P.S.; Nikhil Karthik, R.; Prasad, A.; Dhananjaya, B.L.; Satish, S.; Prasad, H.; Nagendra Prasad, N.N. Phytogenic synthesis of nanoparticles from *Rhizophora mangle* and their bactericidal potential with DNA damage activity. *Nano Struct. Nano Objects* **2017**, *10*, 112–115. doi:10.1016/j.nanoso.2017.03.011.
14. Pandurangan, M.; Nagajyothi, P.C.; Kim, D.H.; Jung, M.J.; Shim, J.; Eom, I.Y. Green synthesis and characterization of biologically active silver nanoparticles using *Perilla frutescens* leaf extract. *J. Clust. Sci.* **2017**, *28*, 81–90. doi:10.1007/s10876-016-1046-3.
15. Jha, D.; Thiruveedula, P.K.; Pathak, R.; Kumar, B.; Gautam, H.K.; Agnihotri, S.; Sharma, A.K.; Kumar, P. Multifunctional biosynthesized silver nanoparticles exhibiting excellent antimicrobial potential against multi-drug resistant microbes along with remarkable anticancerous properties. *Mater. Sci. Eng. C* **2017**, *80*, 659–669. doi:10.1016/j.msec.2017.07.011.
16. Fierascu, I.; Georgiev, M.I.; Ortan, A.; Fierascu, R.C.; Avramescu, S.M.; Ionescu, D.; Sutan, A.; Brinzan, A.; Ditu, L.M. Phyto-mediated metallic nano-architectures via *Melissa officinalis* L.: Synthesis, characterization and biological properties. *Sci. Rep.* **2017**, *7*, 12428.

17. Verma, S.K.; Jha, E.; Sahoo, B.; Panda, P.K.; Thirumurugan, A.; Parashar, S.K.S.; Suar, M. Mechanistic insight into the rapid one-step facile biofabrication of antibacterial silver nanoparticles from bacterial release and their biogenicity and concentration-dependent in vitro cytotoxicity to colon cells. *RSC Adv.* **2017**, *7*, 40034–40045. doi:10.1039/c7ra05943d.
18. Prasad, K.S.; Amin, T.; Katuva, S.; Kumari, M.; Selvaraj, K. Synthesis of water soluble CdS nanoparticles and study of their DNA damage activity. *Arab. J. Chem.* **2017**, *10*, S3929–S3935. doi:10.1016/j.arabjc.2014.05.033.
19. Syed Ali, M.; Anuradha, V.; Sripriya, V.; Yogananth, N.; Sheeba, H. Evaluation of anti-genotoxicity of THE Tio2 nanoparticle biosynthesized from *Turbinaria conoides*. *Int. J. Curr. Res.* **2017**, *9*, 59085–59091.
20. Saha, N.; Dutta Gupta, S. Low-dose toxicity of biogenic silver nanoparticles fabricated by Swertia chirata on root tips and flower buds of Allium cepa. *J. Hazard. Mater.* **2017**, *330*, 18–28. doi:10.1016/j.jhazmat.2017.01.021.
21. Panda, K.K.; Golari, D.; Venugopal, A.; Achary, V.M.M.; Phaomei, G.; Parinandi, N.L.; Sahu, H.K.; Panda, B.B. Green Synthesized Zinc Oxide (ZnO) Nanoparticles Induce Oxidative Stress and DNA Damage in *Lathyrus sativus* L. Root Bioassay System. *Antioxidants* **2017**, *6*, 35. doi:10.3390/antiox6020035.
22. Moteriya, P.; Chanda, S. Synthesis and characterization of silver nanoparticles using *Caesalpinia pulcherrima* flower extract and assessment of their in vitro antimicrobial, antioxidant, cytotoxic, and genotoxic activities. *Artif. Cells Nanomed. Biotechnol.* **2017**, *45*, 1556–1567. doi:10.1080/21691401.2016.1261871.
23. Datkhile, K.D.; Durgawale, P.P.; Patil, M.N. Biogenic silver nanoparticles are equally cytotoxic as chemically synthesized silver nanoparticles. *Biomed. Pharmacol. J.* **2017**, *10*, 337–344. doi:10.13005/bpj/1114.
24. Guilger, M.; Pasquoto-Stigliani, T.; Bilesky-Jose, N.; Grillo, R.; Abhilash, P.C.; Fraceto, L.F.; Lima, R. Biogenic silver nanoparticles based on *Trichoderma harzianum*: Synthesis, characterization, toxicity evaluation and biological activity. *Sci. Rep.* **2017**, *7*, 44421. doi:10.1038/srep44421.
25. Barua, S.; Banerjee, P.P.; Sadhu, A.; Sengupta, A.; Chatterjee, S.; Sarkar, S.; Barman, S.; Chattopadhyay, A.; Battacharya, S.; Mondal, N.C.; et al. Silver nanoparticles as antibacterial and anticancer materials against human breast, cervical and oral cancer cells. *J. Nanosci. Nanotechnol* .**2017**, *17*, 968–976.
26. Bhanumathi, R.; Vimala, K.; Shanthi, K.; Thangaraj, R.; Kannan, S. Bioformulation of silver nanoparticles as berberine carrier cum anticancer agent against breast cancer. *New J. Chem.* **2017**, *41*, 14466–14477. doi:10.1039/C7NJ02531A.
27. Das, P.; Chetia, B.; Prasanth, R.; Madhavan, J.; Singaravelu, G.; Benelli, G.; Murugan, K. Green nanosynthesis and functionalization of gold nanoparticles as PTP 1B inhibitors. *J. Clust. Sci.* **2017**, *28*, 2269–2277. doi:10.1007/s10876-017-1224-y.
28. Baghbani-Arani, F.; Movagharnia, R.; Sharifian, A.; Salehi, S.; Shandiz, S.A.S. Photo-catalytic, anti-bacterial, and anti-cancer properties of phyto-mediated synthesis of silver nanoparticles from *Artemisia tournefortiana* Rchb extract. *J. Photochem. Photobiol. B Biol.* **2017**, *173*, 640–649. doi:10.1016/j.jphotobiol.2017.07.003.
29. Banerjee, P.P.; Bandyopadhyay, A.; Harsha, S.N.; Policegoudra, R.S.; Bhattacharya, S.; Karak, N.; Chattopadhyay, A. *Mentha arvensis* (Linn.)-mediated green silver nanoparticles trigger caspase 9-dependent cell death in MCF7 and MDA-MB-231 cells. *Breast Cancer Targets Ther.* **2017**, *9*, 265–278. doi:10.2147/BCTT.S130952.
30. Khalid, M.; Khalid, N.; Ahmed, I.; Hanif, R.; Ismail, M.; Janjua, H.A. Comparative studies of three novel freshwater microalgae strains for synthesis of silver nanoparticles: Insights of characterization, antibacterial, cytotoxicity and antiviral activities. *J. Appl. Phycol.* **2017**, *29*, 1851–1863. doi:10.1007/s10811-017-1071-0.
31. Datkhile, K.D.; Durgavale, P.P.; Patil, M.N. Biogenic silver nanoparticles from *Nothapodytes foetida* kills human cancer cells In vitro through inhibition of cell proliferation and induction of apoptosis. *J. Bionanoscience* **2017**, *11*, 416–427. doi:10.1166/jbns.2017.1465.
32. Chandrakasan, G.; Seetharaman, P.; Gnanasekar, S.; Kadarkarai, M.; Sivaperumal, S. Xenorhabdus stockiae KT835471-mediated feasible biosynthesis of metal nanoparticles for their antibacterial and cytotoxic activities. *Artif. Cells Nanomed. Biotechnol.* **2017**, *45*, 1675–1684. doi:10.1080/21691401.2017.1282495.
33. Das Nelaturi, P.; Sriramaia, N.H.; Nagaraj, S.; Kotakadi, V.S.; Moideen Kutty, A.V.V.; Pamidimukkala, K. An in-vitro cytotoxic and genotoxic properties of *Allamanda cathartica* L. latex green NPs on human peripheral blood mononuclear cells. *Nano Biomed. Eng.* **2017**, *9*, 314–323.

34. Daphedar, A.; Taranath, T.C. Biosynthesis of silver nanoparticles by leaf extract of *Albizia saman* (Jacq.) Merr. and their cytotoxic effect on mitotic chromosomes of *Drimia indica* (Roxb.) Jessop. *Environ. Sci. Pollut. Res. Int.* **2017**, *24*, 25861–25869. doi:10.1007/s11356-017-9899-z.
35. Şuţan, N.A.; Fierăscu, I.; Fierăscu, R.C.; Manolescu, D.T.; Soare, L.C. Comparative analytical characterization and in vitro cytogenotoxic activity evaluation of *Asplenium scolopendrium* L. leaves and rhizome extracts prior to and after Ag nanoparticles phytosynthesis. *Ind. Crop. Prod.* **2016**, *83*, 379–386. doi:10.1016/j.indcrop.2016.01.011.
36. Mishra, P.; Ray, S.; Sinha, S.; Das, B.; Khan, M.I.; Behera, S.K.; Yun, S.I.; Tripathy, S.K.; Mishra, A. Facile bio-synthesis of gold nanoparticles by using extract of *Hibiscus sabdariffa* and evaluation of its cytotoxicity against U87 glioblastoma cells under hyperglycemic condition. *Biochem. Eng. J.* **2016**, *105*, 264–272. doi:10.1016/j.bej.2015.09.021.
37. Qi, L.; Lv, X.; Zhang, T.; Jia, P.; Yan, R.; Li, S.; Zou, R.; Xue, Y.; Dai, L. Cytotoxicity and genotoxicity of bacterial magnetosomes against human retinal pigment epithelium cells. *Sci. Rep.* **2016**, *6*, 26961. doi:10.1038/srep26961.
38. Perde-Schrepler, M.; David, L.; Olenic, L.; Potara, M.; Fischer-Fodor, E.; Virag, P.; Imre-Lucaci, F.; Brie, I.; Florea, A. Gold nanoparticles synthesized with a polyphenols-rich extract from cornelian cherry (*Cornus mas*) fruits: Effects on human skin cells. *J. Nanomater.* **2016**, *2016*, 6986370. doi:10.1155/2016/6986370.
39. KS, U.S.; Govindaraju, K.; Kumar, G.; Prabhu, D.; Arulvasu, C.; Karthick, V.; Changmai, N. Anti-proliferative effect of biogenic gold nanoparticles against breast cancer cell lines (MDA-MB-231 & MCF-7). *Appl. Surf. Sci.* **2016**, *371*, 415–424. doi:10.1016/j.apsusc.2016.03.004.
40. Suganya, K.S.U.; Govindaraju, K.; Kumar, V.G.; Karthick, V.; Parthasarathy, K. Pectin mediated gold nanoparticles induces apoptosis in mammary adenocarcinoma cell lines. *Int. J. Biol. Macromol.* **2016**, *93*, 1030–1040. doi:10.1016/j.ijbiomac.2016.08.086.
41. Panda, K.K.; Achary, V.M.; Phaomie, G.; Sahu, H.K.; Parinandi, N.L.; Panda, B.B. Polyvinyl polypyrrolidone attenuates genotoxicity of silver nanoparticles synthesized via green route, tested in *Lathyrus sativus* L. root bioassay. *Mutat. Res. Genet. Toxicol. Environ. Mutagenesis* **2016**, *806*, 11–23. doi:10.1016/j.mrgentox.2016.05.006.
42. Farah, M.A.; Ali, M.A.; Chen, S.M.; Li, Y.; Al-Hemaid, F.M.; Abou-Tarboush, F.M.; Al-Anazi, K.M.; Lee, J. Silver nanoparticles synthesized from *Adenium obesum* leaf extract induced DNA damage, apoptosis and autophagy via generation of reactive oxygen species. *Colloids Surf. B Biointerfaces* **2016**, *141*, 158–169. doi:10.1016/j.colsurfb.2016.01.027.
43. Elshawy, O.E.; Helmy, E.A.; Rashed, L.A. Preparation, characterization and in vitro evaluation of the antitumor activity of the biologically synthesized silver nanoparticles. *Adv. Nanoparticles* **2016**, *5*, 149. doi:10.4236/anp.2016.52017.
44. Jang, S.J.; Yang, I.J.; Tettey, C.O.; Kim, K.M.; Shin, H.M. In-vitro anticancer activity of green synthesized silver nanoparticles on MCF-7 human breast cancer cells. *Mater. Sci. Eng. C* **2016**, *68*, 430–435. doi:10.1016/j.msec.2016.03.101.
45. Prabhu, A.; Shankar, K.; Muthukrishnan, P.; Kathiresan, A.; Prakash, P. An investigation on the cytotoxicity and apoptotic effect of biologically synthesized silver nanoparticles on MCF-7 and A549 cell lines using weed *Setaria verticillata* L. *Indo Am. J. Pharm. Sci.* **2016**, *3*, 37–43.
46. Prasannaraj, G.; Sahi, S.V.; Ravikumar, S.; Venkatachalam, P. Enhanced cytotoxicity of biomolecules loaded metallic silver nanoparticles against human liver (HepG2) and prostate (PC3) cancer cell lines. *J. Nanosci. Nanotechnol.* **2016**, *16*, 4948–4959. doi:10.1166/jnn.2016.12336.
47. Kayalvizhi, T.; Ravikumar, S.; Venkatachalam, P. Green synthesis of metallic silver nanoparticles using *Curculigo orchioides* rhizome extracts and evaluation of its antibacterial, larvicidal, and anticancer activity. *J. Environ. Eng.* **2016**, *142*, C4016002. doi:10.1061/(asce)ee.1943¨C7870.0001098.
48. Selvi, B.C.G.; Madhavan, J.; Amutha, S. Cytotoxic effect of silver nanoparticles synthesized from *Padina tetrastromatica* on breast cancer cell line. *Adv. Nat. Sci. Nanosci. Nanotechnol.* **2016**, *7*, 035015.
49. Chandramohan, B.; Murugan, K.; Panneerselvam, C.; Madhiyazhagan, P.; Chandirasekar, R.; Dinesh, D.; Kumar, P.M.; Kovendan, K.; Suresh, U.; Subramaniam, J.; et al. Characterization and mosquitocidal potential of neem cake-synthesized silver nanoparticles: Genotoxicity and impact on predation efficiency of mosquito natural enemies. *Parasitol. Res.* **2016**, *115*, 1015–1025. doi:10.1007/s00436-015-4829-9.

50. Kajani, A.A.; Zarkesh-Esfahani, S.H.; Bordbar, A.K.; Khosropour, A.R.; Razmjou, A.; Kardi, M. Anticancer effects of silver nanoparticles encapsulated by *Taxus baccata* extracts. *J. Mol. Liq.* **2016**, *223*, 549–556. doi:10.1016/j.molliq.2016.08.064.
51. Kalangi, S.K.; Dayakar, A.; Gangappa, D.; Sathyavathi, R.; Maurya, R.S.; Narayana Rao, D. Biocompatible silver nanoparticles reduced from *Anethum graveolens* leaf extract augments the antileishmanial efficacy of miltefosine. *Exp. Parasitol.* **2016**, *170*, 184–192. doi:10.1016/j.exppara.2016.09.002.
52. He, Y.; Du, Z.; Ma, S.; Cheng, S.; Jiang, S.; Liu, Y.; Li, D.; Huang, H.; Zhang, K.; Zheng, X. Biosynthesis, antibacterial activity and anticancer effects against prostate cancer (PC-3) cells of silver nanoparticles using *Dimocarpus longan* lour. peel extract. *Nanoscale Res. Lett.* **2016**, *11*, 300. doi:10.1186/s11671-016-1511-9.
53. Bhakya, S.; Muthukrishnan, S.; Sukumaran, M.; Grijalva, M.; Cumbal, L.; Franklin Benjamin, J.H.; Senthil Kumar, T.; Rao, M.V. Antimicrobial, antioxidant and anticancer activity of biogenic silver nanoparticles—An experimental report. *RSC Adv.* **2016**, *6*, 81436–81446. doi:10.1039/C6RA17569D.
54. Iram, S.; Khan, S.; Ansary, A.A.; Arshad, M.; Siddiqui, S.; Ahmad, E.; Khan, R.H.; Khan, M.S. Biogenic terbium oxide nanoparticles as the vanguard against osteosarcoma. *Spectrochim. Acta Part A Mol. Biomol. Spectrosc.* **2016**, *168*, 123–131. doi:10.1016/j.saa.2016.05.053.
55. Mata, R.; Nakkala, J.R.; Sadras, S.R. Polyphenol stabilized colloidal gold nanoparticles from *Abutilon indicum* leaf extract induce apoptosis in HT-29 colon cancer cells. *Colloids Surf. B Biointerfaces* **2016**, *143*, 499–510. doi:10.1016/j.colsurfb.2016.03.069.
56. Azmath, P.; Baker, S.; Rakshith, D.; Satish, S. Mycosynthesis of silver nanoparticles bearing antibacterial activity. *Saudi Pharm. J.* **2016**, *24*, 140–146. doi:10.1016/j.jsps.2015.01.008.
57. Ashe, S.; Nayak, D.; Kumari, M.; Nayak, B. Ameliorating effects of green synthesized silver nanoparticles on glycated end product induced reactive oxygen species production and cellular toxicity in osteogenic Saos-2 cells. *ACS Appl. Mater. Interfaces* **2016**, *8*, 30005–30016. doi:10.1021/acsami.6b10639.
58. Balashanmugam, P.; Durai, P.; Balakumaran, M.D.; Kalaichelvan, P.T. Phytosynthesized gold nanoparticles from *C. roxburghii* DC. leaf and their toxic effects on normal and cancer cell lines. *J. Photochem. Photobiol. Biol.* **2016**, *165*, 163–173. doi:10.1016/j.jphotobiol.2016.10.013.
59. Balaji, K.; Gothandam, K.M. Cytotoxic effect on cancerous cell lines by biologically synthesized silver nanoparticles. *Braz. Arch. Biol. Technol.* **2016**, *59*. doi:10.1590/1678-4324-2016150529.
60. Qayyum, S.; Khan, A.U. Biofabrication of broad range antibacterial and antibiofilm silver nanoparticles. *IET Nanobiotechnol.* **2016**, *10*, 349–357. doi:10.1049/iet-nbt.2015.0091.
61. Thiruvengadam, M.; Gurunathan, S.; Chung, I.M. Physiological, metabolic, and transcriptional effects of biologically-synthesized silver nanoparticles in turnip (*Brassica rapa* ssp. *rapa* L.). *Protoplasma* **2015**, *252*, 1031–1046. doi:10.1007/s00709-014-0738-5.
62. Parandhaman, T.; Das, A.; Ramalingam, B.; Samanta, D.; Sastry, T.P.; Mandal, A.B.; Das, S.K. Antimicrobial behavior of biosynthesized silica-silver nanocomposite for water disinfection: A mechanistic perspective. *J. Hazard. Mater.* **2015**, *290*, 117–126. doi:10.1016/j.jhazmat.2015.02.061.
63. Jeyaraj, M.; Renganathan, A.; Sathishkumar, G.; Ganapathi, A.; Premkumar, K. Biogenic metal nanoformulations induce Bax/Bcl2 and caspase mediated mitochondrial dysfunction in human breast cancer cells (MCF 7). *RSC Adv.* **2015**, *5*, 2159–2166. doi:10.1039/c4ra11686k.
64. Mata, R.; Nakkala, J.R.; Sadras, S.R. Biogenic silver nanoparticles from *Abutilon indicum*: Their antioxidant, antibacterial and cytotoxic effects in vitro. *Colloids Surf. B Biointerfaces* **2015**, *128*, 276–286. doi:10.1016/j.colsurfb.2015.01.052.
65. Zahir, A.A.; Chauhan, I.S.; Bagavan, A.; Kamaraj, C.; Elango, G.; Shankar, J.; Arjaria, N.; Roopan, S.M.; Rahuman, A.A.; Singh, N. Green synthesis of silver and titanium dioxide nanoparticles using *Euphorbia prostrata* extract shows shift from apoptosis to G0/G1 arrest followed by necrotic cell death in *Leishmania donovani*. *Antimicrob. Agents Chemother.* **2015**, *59*, 4782–4799. doi:10.1128/AAC.00098-15.
66. Manna, D.K.; Mandal, A.K.; Sen, I.K.; Maji, P.K.; Chakraborti, S.; Chakraborty, R.; Islam, S.S. Antibacterial and DNA degradation potential of silver nanoparticles synthesized via green route. *Int. J. Biol. Macromol.* **2015**, *80*, 455–459. doi:10.1016/j.ijbiomac.2015.07.028.
67. Subbaiya, R.; Masilamani Selvam, M.; Sundar, K. Biological synthesis of silver nanorods from *Nocardia mediterranei*-5016 and its antitumor activity against non-small cell lung carcinoma cell line. *Int. J. PharmTech Res.* **2015**, *8*, 298–307.

68. Gandhiraj, V.; Sathish Kumar, K.; Madhusudhanan, J.; Sandhya, J. Antitumor activity of biosynthesized silver nano particles from leaves of *Momordica charantia* against MCF-7 cell line. *Int. J. ChemTech Res.* **2015**, *8*, 351–362.
69. Dwivedi, S.; Saquib, Q.; Al-Khedhairy, A.A.; Ahmad, J.; Siddiqui, M.A.; Musarrat, J. Rhamnolipids functionalized AgNPs-induced oxidative stress and modulation of toxicity pathway genes in cultured MCF-7 cells. *Colloids Surf. B Biointerfaces* **2015**, *132*, 290–298. doi:10.1016/j.colsurfb.2015.05.034.
70. Baskar, V.; Venkatesh, J.; Park, S.W. Impact of biologically synthesized silver nanoparticles on the growth and physiological responses in *Brassica rapa* ssp. pekinensis. *Environ. Sci. Pollut. Res.* **2015**, *22*, 17672–17682. doi:10.1007/s11356-015-4864-1.
71. Chung, I.M.; Rahuman, A.A.; Marimuthu, S.; Kirthi, A.V.; Anbarasan, K.; Rajakumar, G. An investigation of the cytotoxicity and caspase-mediated apoptotic effect of green synthesized zinc oxide nanoparticles using *Eclipta prostrata* on human liver carcinoma cells. *Nanomaterials* **2015**, *5*, 1317–1330. doi:10.3390/nano5031317.
72. Baharara, J.; Namvar, F.; Ramezani, T.; Mousavi, M.; Mohamad, R. Silver nanoparticles biosynthesized using *Achillea biebersteinii* flower extract: Apoptosis induction in mcf-7 cells via caspase activation and regulation of Bax and Bcl-2 gene expression. *Molecules* **2015**, *20*, 2693–2706.
73. Ramar, M.; Manikandan, B.; Marimuthu, P.N.; Raman, T.; Mahalingam, A.; Subramanian, P.; Karthick, S.; Munusamy, A. Synthesis of silver nanoparticles using *Solanum trilobatum* fruits extract and its antibacterial, cytotoxic activity against human breast cancer cell line MCF 7. *Spectrochim. Acta Part A Mol. Biomol. Spectrosc.* **2015**, *140*, 223–228. doi:10.1016/j.saa.2014.12.060.
74. Gurunathan, S.; Park, J.H.; Han, J.W.; Kim, J.H. Comparative assessment of the apoptotic potential of silver nanoparticles synthesized by *Bacillus tequilensis* and *Calocybe indica* in MDA-MB-231 human breast cancer cells: Targeting p53 for anticancer therapy. *Int. J. Nanomed.* **2015**, *10*, 4203–4223. doi:10.2147/IJN.S83953.
75. Gurunathan, S.; Jeong, J.K.; Han, J.W.; Zhang, X.F.; Park, J.H.; Kim, J.H. Multidimensional effects of biologically synthesized silver nanoparticles in *Helicobacter pylori*, *Helicobacter felis*, and human lung (L132) and lung carcinoma A549 cells. *Nanoscale Res. Lett.* **2015**, *10*, 1–17. doi:10.1186/s11671-015-0747-0.
76. Ismail, A.F.M.; Ahmed, M.M.; Salem, A.A.M. Biosynthesis of silver nanoparticles using mushroom extracts: Induction of apoptosis in HepG2 and MCF-7 Cells via caspases stimulation and regulation of BAX and Bcl-2 gene expressions. *J. Pharm. Biomed. Sci.* **2015**, *5*, 1–9.
77. Namvar, F.; Rahman, H.S.; Mohamad, R.; Azizi, S.; Tahir, P.M.; Chartrand, M.S.; Yeap, S.K. Cytotoxic effects of biosynthesized zinc oxide nanoparticles on murine cell lines. *Evid. Based Complementary Altern. Med.* **2015**, *2015*, 593014.
78. Parveen, A.; Rao, S. Cytotoxicity and genotoxicity of biosynthesized gold and silver nanoparticles on human cancer cell lines. *J. Clust. Sci.* **2015**, *26*, 775–788. doi:10.1007/s10876-014-0744-y.
79. Ramar, M.; Manikandan, B.; Raman, T.; Arunagirinathan, K.; Prabhu, N.M.; Basu, M.J.; Perumal, M.; Palanisamy, S.; Munusamy, A. Biosynthesis of silver nanoparticles using ethanolic petals extract of *Rosa indica* and characterization of its antibacterial, anticancer and anti-inflammatory activities. *Spectrochim. Acta Part A Mol. Biomol. Spectrosc.* **2015**, *138*, 120–129. doi:10.1016/j.saa.2014.10.043.
80. Krishnaraj, C.; Harper, S.L.; Choe, H.S.; Kim, K.P.; Yun, S.I. Mechanistic aspects of biologically synthesized silver nanoparticles against food- and water-borne microbes. *Bioprocess Biosyst. Eng.* **2015**, *38*, 1943–1958. doi:10.1007/s00449-015-1436-1.
81. Hullikere, M.M.; Joshi, C.G.; Vijay, R.; Ananda, D.; Nivya, M. Antiangiogenic, cytotoxic and antimicrobial activity of plant mediated silver nano particle from *Tragia involucrate*. *Res. J. Nanosci. Nanotechnol.* **2015**, *5*, 16–26.
82. Govindaraju, K.; Krishnamoorthy, K.; Alsagaby, S.A.; Singaravelu, G.; Premanathan, M. Green synthesis of silver nanoparticles for selective toxicity towards cancer cells. *LET Nanobiotechnol.* **2015**, *9*, 325–330. doi:10.1049/iet-nbt.2015.0001.
83. Ortega, F.G.; Fernández-Baldo, M.A.; Fernández, J.G.; Serrano, M.J.; Sanz, M.I.; Diaz-Mochón, J.J.; Lorente, J.A.; Raba, J. Study of antitumor activity in breast cell lines using silver nanoparticles produced by yeast. *Int. J. Nanomed.* **2015**, *10*, 2021–2031. doi:10.2147/IJN.S75835.
84. Raman, J.; Reddy, G.R.; Lakshmanan, H.; Selvaraj, V.; Gajendran, B.; Nanjian, R.; Chinnasamy, A.; Sabaratnam, V. Mycosynthesis and characterization of silver nanoparticles from *Pleurotus djamor* var.

roseus and their in vitro cytotoxicity effect on PC3 cells. *Process Biochem.* **2015**, *50*, 140–147. doi:10.1016/j.procbio.2014.11.003.

85. Vijaya, P.P.; Rekha, B.; Mathew, A.T.; Ali, M.S.; Yogananth, N.; Anuradha, V.; Parveen, P.K. Antigenotoxic effect of green-synthesised silver nanoparticles from *Ocimum sanctum* leaf extract against cyclophosphamide induced genotoxicity in human lymphocytes-in vitro. *Appl. Nanosci.* **2014**, *4*, 415–420. doi:10.1007/s13204-013-0212-2.
86. Rajasekharreddy, P.; Rani, P.U. Biofabrication of Ag nanoparticles using *Sterculia foetida* L. seed extract and their toxic potential against mosquito vectors and HeLa cancer cells. *Mater. Sci. Eng. C* **2014**, *39*, 203–212. doi:10.1016/j.msec.2014.03.003.
87. Krishnasamy, L. Cytotoxic, apoptotic efficacy of silver nanoparticles synthesized from *Indigofera aspalathoids*. *Int. J. Pharm. Pharm. Sci.* **2014**, *6*, 245–248.
88. Prasad, K.S.; Selvaraj, K. Biogenic synthesis of selenium nanoparticles and their effect on as(III)-induced toxicity on human lymphocytes. *Biol. Trace Elem. Res.* **2014**, *157*, 275–283. doi:10.1007/s12011-014-9891-0.
89. Sarkar, J.; Ghosh, M.; Mukherjee, A.; Chattopadhyay, D.; Acharya, K. Biosynthesis and safety evaluation of ZnO nanoparticles. *Bioprocess Biosyst. Eng.* **2014**, *37*, 165–171. doi:10.1007/s00449-013-0982-7.
90. Kumar, S.; Singh, M.; Halder, D.; Mitra, A. Mechanistic study of antibacterial activity of biologically synthesized silver nanocolloids. *Colloids Surf. A Physicochem. Eng. Asp.* **2014**, *449*, 82–86. doi:10.1016/j.colsurfa.2014.02.027.
91. El-Kassas, H.Y.; El-Sheekh, M.M. Cytotoxic activity of biosynthesized gold nanoparticles with an extract of the red seaweed *Corallina officinalis* on the MCF-7 human breast cancer cell line. *Asian Pac. J. Cancer Prev.* **2014**, *15*, 4311–4317.
92. Chowdhury, S.; Basu, A.; Kundu, S. Green synthesis of protein capped silver nanoparticles from phytopathogenic fungus *Macrophomina phaseolina* (Tassi) Goid with antimicrobial properties against multidrug-resistant bacteria. *Nanoscale Res. Lett.* **2014**, *9*, 365. doi:10.1186/1556-276x-9-365.
93. Krishnaraj, C.; Muthukumaran, P.; Ramachandran, R.; Balakumaran, M.D.; Kalaichelvan, P.T. *Acalypha indica* Linn: Biogenic synthesis of silver and gold nanoparticles and their cytotoxic effects against MDA-MB-231, human breast cancer cells. *Biotechnol. Rep.* **2014**, *4*, 42–49. doi:10.1016/j.btre.2014.08.002.
94. Lima, R.; Feitosa, L.O.; Ballottin, D.; Marcato, P.D.; Tasic, L.; Duran, N. Cytotoxicity and genotoxicity of biogenic silver nanoparticles. *J. Phys.: Conf. Ser.* **2013**, 429, 012020.
95. Singh, G.; Babele, P.K.; Shahi, S.K.; Sinha, R.P.; Tyagi, M.B.; Kumar, A. Green synthesis of silver nanoparticles using cell extracts of *Anabaena doliolum* and screening of its antibacterial and antitumor activity. *J. Microbiol. Biotechnol.* **2014**, *24*, 1354–1367.
96. Varun, S.; Sellappa, S. Enhanced apoptosis in MCF-7 human breast cancer cells by biogenic gold nanoparticles synthesized from Argemone mexicana leaf extract. *Int. J. Pharm. Pharm. Sci.* **2014**, *6*, 528–531.
97. Subbaiya, R.; Selvam, M. Synthesis and characterization of silver nanoparticles from *Streptomyces olivaceus* sp-1392 and its anticancerous activity against non-small cell lung carcinoma cell line (NCI-H460). *Curr. Nanosci.* **2014**, *10*, 243–249. doi:10.2174/15734137113099990073.
98. Ashokkumar, T.; Prabhu, D.; Geetha, R.; Govindaraju, K.; Manikandan, R.; Arulvasu, C.; Singaravelu, G. Apoptosis in liver cancer (HepG2) cells induced by functionalized gold nanoparticles. *Colloids Surf. B Biointerfaces* **2014**, *123*, 549–556. doi:10.1016/j.colsurfb.2014.09.051.
99. Jeyaraj, M.; Arun, R.; Sathishkumar, G.; Mubarakali, D.; Rajesh, M.; Sivanandhan, G.; Kapildev, G.; Manickavasagam, M.; Thajuddin, N.; Ganapathi, A. An evidence on G2/M arrest, DNA damage and caspase mediated apoptotic effect of biosynthesized gold nanoparticles on human cervical carcinoma cells (HeLa). *Mater. Res. Bull.* **2014**, *52*, 15–24. doi:10.1016/j.materresbull.2013.12.060.
100. Prasad, K.S.; Patel, H.; Patel, T.; Patel, K.; Selvaraj, K. Biosynthesis of Se nanoparticles and its effect on UV-induced DNA damage. *Colloids Surf. Bbiointerfaces* **2013**, *103*, 261–266. doi:10.1016/j.colsurfb.2012.10.029.
101. Rosarin, F.S.; Arulmozhi, V.; Nagarajan, S.; Mirunalini, S. Antiproliferative effect of silver nanoparticles synthesized using amla on Hep2 cell line. *Asian Pac. J. Trop. Med.* **2013**, *6*, 1–10. doi:10.1016/S1995-7645(12)60193-X.
102. Neveen, M.K. Biogenic silver nanoparticles by *Aspergillus terreus* as a powerful nanoweapon against *Aspergillus fumigatus*. *Afr. J. Microbiol. Res.* **2013**, *7*, 5645–5651. doi:10.5897/AJMR2013.6429.

103. Mohanty, S.; Jena, P.; Mehta, R.; Pati, R.; Banerjee, B.; Patil, S.; Sonawane, A. Cationic antimicrobial peptides and biogenic silver nanoparticles kill mycobacteria without eliciting DNA damage and cytotoxicity in mouse macrophages. *Antimicrob. Agents Chemother.* **2013**, *57*, 3688–3698. doi:10.1128/aac.02475-12.
104. Jeyaraj, M.; Sathishkumar, G.; Sivanandhan, G.; MubarakAli, D.; Rajesh, M.; Arun, R.; Kapildev, G.; Manickavasagam, M.; Thajuddin, N.; Premkumar, K.; et al. Biogenic silver nanoparticles for cancer treatment: An experimental report. *Colloids Surf. B Biointerfaces* **2013**, *106*, 86–92. doi:10.1016/j.colsurfb.2013.01.027.
105. Jeyaraj, M.; Rajesh, M.; Arun, R.; MubarakAli, D.; Sathishkumar, G.; Sivanandhan, G.; Dev, G.K.; Manickavasagam, M.; Premkumar, K.; Thajuddin, N.; et al. An investigation on the cytotoxicity and caspase-mediated apoptotic effect of biologically synthesized silver nanoparticles using *Podophyllum hexandrum* on human cervical carcinoma cells. *Colloids Surf. B Biointerfaces* **2013**, *102*, 708–717. doi:10.1016/j.colsurfb.2012.09.042.
106. Prabhu, D.; Arulvasu, C.; Babu, G.; Manikandan, R.; Srinivasan, P. Biologically synthesized green silver nanoparticles from leaf extract of *Vitex negundo* L. induce growth-inhibitory effect on human colon cancer cell line HCT15. *Process Biochem.* **2013**, *48*, 317–324. doi:10.1016/j.procbio.2012.12.013.
107. Geetha, R.; Ashokkumar, T.; Tamilselvan, S.; Govindaraju, K.; Sadiq, M.; Singaravelu, G. Green synthesis of gold nanoparticles and their anticancer activity. *Cancer Nanotechnol.* **2013**, *4*, 91–98. doi:10.1007/s12645-013-0040-9.
108. Govender, R.; Phulukdaree, A.; Gengan, R.M.; Anand, K.; Chuturgoon, A.A. Silver nanoparticles of *Albizia adianthifolia*: The induction of apoptosis in human lung carcinoma cell line. *J. Nanobiotechnol.* **2013**, *11*, 5. doi:10.1186/1477-3155-11-5.
109. Chunyan, W.; Valiyaveettil, S. Correlation of biocapping agents with cytotoxic effects of silver nanoparticles on human tumor cells. *RSC Adv.* **2013**, *3*, 14329–14338. doi:10.1039/c3ra41346b.
110. Gurunathan, S.; Raman, J.; Malek, N.A.; John, P.A.; Vikineswary, S. Green synthesis of silver nanoparticles using *Ganoderma neo-japonicum* Imazeki: A potential cytotoxic agent against breast cancer cells. *Int. J. Nanomed.* **2013**, *8*, 4399–4413. doi:10.2147/ijn.s51881.
111. Gurunathan, S.; Han, J.W.; Eppakayala, V.; Jeyaraj, M.; Kim, J.H. Cytotoxicity of biologically synthesized silver nanoparticles in MDA-MB-231 human breast cancer cells. *Biomed. Res. Int.* **2013**, *2013*. doi:10.1155/2013/535796.
112. Wu, H.; Zhu, H.; Li, X.; Liu, Z.; Zheng, W.; Chen, T.; Yu, B.; Wong, K.H. Induction of apoptosis and cell cycle arrest in A549 human lung adenocarcinoma cells by surface-capping selenium nanoparticles: An effect enhanced by polysaccharide-protein complexes from *Polyporus rhinocerus*. *J. Agric. Food Chem.* **2013**, *61*, 9859–9866. doi:10.1021/jf403564s.
113. Gurunathan, S.; Han, J.W.; Dayem, A.A.; Eppakayala, V.; Park, J.H.; Cho, S.G.; Lee, K.J.; Kim, J.H. Green synthesis of anisotropic silver nanoparticles and its potential cytotoxicity in human breast cancer cells (MCF-7). *J. Ind. Eng. Chem.* **2013**, *19*, 1600–1605. doi:10.1016/j.jiec.2013.01.029.
114. Tamboli, D.P.; Lee, D.S. Mechanistic antimicrobial approach of extracellularly synthesized silver nanoparticles against gram positive and gram negative bacteria. *J. Hazard. Mater.* **2013**, *260*, 878–884. doi:10.1016/j.jhazmat.2013.06.003.
115. Das, S.; Das, J.; Samadder, A.; Bhattacharyya, S.S.; Das, D.; Khuda-Bukhsh, A.R. Biosynthesized silver nanoparticles by ethanolic extracts of *Phytolacca decandra, Gelsemium sempervirens*, *Hydrastis canadensis* and *Thuja occidentalis* induce differential cytotoxicity through G2/M arrest in A375 cells. *Colloids Surf. B Biointerfaces* **2013**, *101*, 325–336. doi:10.1016/j.colsurfb.2012.07.008.
116. Bhattacharyya, S.S.; Das, J.; Das, S.; Samadder, A.; Das, D.; De, A.; Paul, S.; Khuda-Bukhsh, A.R. Rapid green synthesis of silver nanoparticles from silver nitrate by a homeopathic mother tincture *Phytolacca decandra*. *J. Chin. Integr. Med.* **2012**, *10*, 546–554. doi:10.3736/jcim20120510.
117. Mishra, A.; Mehdi, S.J.; Irshad, M.; Ali, A.; Sardar, M.; Rizvi, M.M.A. Effect of biologically synthesized silver nanoparticles on human cancer cells. *Sci. Adv. Mater.* **2012**, *4*, 1200–1206. doi:10.1166/sam.2012.1414.
118. Bendale, Y.; Bendale, V.; Paul, S.; Bhattacharyya, S.S. Green synthesis, characterization and anticancer potential of platinum nanoparticles bioplatin. *J. Chin. Integr. Med.* **2012**, *10*, 681–689. doi:10.3736/jcim20120613.

119. Sarkar, J.; Chattopadhyay, D.; Patra, S.; Deo, S.S.; Sinha, S.; Ghosh, M.; Mukherjee, A.; Acharya, K. *Alternaria alternata* mediated synthesis of protein capped silver nanoparticles and their genotoxic activity. *Dig. J. Nanomater. Biostructures* **2011**, *6*, 563–573.
120. Satyavani, K.; Gurudeeban, S.; Ramanathan, T.; Balasubramanian, T. Biomedical potential of silver nanoparticles synthesized from calli cells of *Citrullus colocynthis* (L.) Schrad. *J. Nanobiotechnol.* **2011**, *9*. doi:10.1186/1477¨C3155¨C9¨C43.
121. Panda, K.K.; Achary, V.M.; Krishnaveni, R.; Padhi, B.K.; Sarangi, S.N.; Sahu, S.N.; Panda, B.B. In vitro biosynthesis and genotoxicity bioassay of silver nanoparticles using plants. *Toxicology* in vitro. *Int. J. Publ. Assoc. Bibra* **2011**, *25*, 1097–1105. doi:10.1016/j.tiv.2011.03.008.
122. Singh, S.; D'Britto, V.; Bharde, A.; Sastry, M.; Dhawan, A.; Prasad, B.L.V. Bacterial synthesis of photocatalytically active and biocompatible TiO_2 and ZnO nanoparticles. *Int. J. Green Nanotechnol. Phys. Chem.* **2010**, *2*, P80–P99. doi:10.1080/19430876.2010.532459.
123. Adiguzel, A.O.; Adiguzel, S.K.; Mazmanci, B.; Tuncer, M.; Mazmanci, M.A. Silver nanoparticle biosynthesis from newly isolated streptomyces genus from soil. *Mater. Res. Express.* **2018**, *5*, 045402. doi:10.1088/2053¨C1591/aab861.
124. Pandiarajan, J.; Balaji, S. Genotoxic effect of silver nanoparticles in silk worm *Bombyx mori*. *Austin J. Biotechnol. Bioeng.* **2018**, *5*, 1096.
125. Gavade, N.L.; Babar, S.B.; Kadam, A.N.; Gophane, A.D.; Garadkar, K.M. Fabrication of M@CuO/ZnO (M = Ag, Au) Heterostructured nanocomposite with enhanced photocatalytic performance under sunlight. *Ind. Eng. Chem. Res.* **2017**, *56*, 14489–14501. doi:10.1021/acs.iecr.7b03168.
126. Ishwarya, R.; Vaseeharan, B.; Shanthi, S.; Ramesh, S.; Manogari, P.; Dhanalakshmi, K.; Vijayakumar, S.; Benelli, G. Green synthesized silver nanoparticles: Toxicity against *Poecilia reticulata* fishes and *Ceriodaphnia cornuta* crustaceans. *J. Clust. Sci.* **2017**, *28*, 519–527. doi:10.1007/s10876-016-1126-4.
127. Krishnaraj, C.; Harper, S.L.; Yun, S.I. In vivo toxicological assessment of biologically synthesized silver nanoparticles in adult Zebrafish (*Danio rerio*). *J. Hazard. Mater.* **2016**, *301*, 480–491. doi:10.1016/j.jhazmat.2015.09.022.
128. Beheshti, N.; Soflaei, S.; Shakibaie, M.; Yazdi, M.H.; Ghaffarifar, F.; Dalimi, A.; Shahverdi, A.R. Efficacy of biogenic selenium nanoparticles against *Leishmania major*: In vitro and in vivo studies. *J. Trace Elem. Med. Biol.* **2013**, *27*, 203–207. doi:10.1016/j.jtemb.2012.11.002.
129. Antony, J.J.; Sithika, M.A.A.; Joseph, T.A.; Suriyakalaa, U.; Sankarganesh, A.; Siva, D.; Kalaiselvi, S.; Achiraman, S. In vivo antitumor activity of biosynthesized silver nanoparticles using *Ficus religiosa* as a nanofactory in DAL induced mice model. *Colloids Surf. B Biointerfaces* **2013**, *108*, 185–190. doi:10.1016/j.colsurfb.2013.02.041.
130. Sukirtha, R.; Krishnan, M.; Ramachandran, R.; Kamalakkannan, S.; Kokilavani, P.; SankarGanesh, D.; Kannan, S.; Achiraman, S. Areca catechu Linn.-derived silver nanoparticles: A novel antitumor agent against Dalton's ascites lymphoma. *Int. J. Green Nanotechnol. Biomed.* **2011**, *3*, 1–12. doi:10.1080/19430892.2011.571626.
